# Conservation, origin, and remodeling of the phenyl acetate degradation pathway

**DOI:** 10.1099/mgen.0.001719

**Published:** 2026-05-19

**Authors:** Jimena Arreola-Calderon, Juan-Manuel Hurtado-Ramirez, Erick Cruz-Santiago, Rosa-Maria Gutierrez-Rios

**Affiliations:** 1 Instituto de Biotecnología, Departamento de Microbiología, Universidad Nacional Autónoma de México, Cuernavaca, C.P. 62210, Morelos, México

**Keywords:** phenylacetic acid hybrid pathway, phenylacetate-CoA ligase, Bacteria, Archaea, genomic marker co-evolution, paralogs

## Abstract

Aromatic hydrocarbons are widespread environmental compounds whose microbial degradation is essential for detoxification, yet the genomic distribution and evolutionary stability of many catabolic pathways remain poorly understood. The phenylacetic acid (PAA) hybrid pathway integrates aerobic and anaerobic features and represents a central route for aromatic compound degradation; however, its prevalence, evolutionary conservation and regulatory organization across prokaryotes have not been comprehensively reassessed.

Here, we investigated the global distribution, structural conservation and evolutionary dynamics of the PAA pathway across prokaryotic genomes. We performed a large-scale comparative genomic analysis of 6,536 bacterial and archaeal genomes, leveraging functionally annotated genomes to enable consistent identification of pathway components, integrating protein architecture profiling, genomic context organization, phylogenetics, motif and structural analyses of phenylacetate-CoA ligase (PAL) and cophylogenetic inference. Using stringent criteria, we identified 853 high-confidence PAL predictions across 825 genomes (≈12%), revealing substantial previously unrecognized PAA metabolic potential. Structural analyses confirmed the conservation of key catalytic features, supporting functional annotation of newly identified PALs. Genomic context analyses revealed extensive pathway remodelling across lineages, yet a conserved core of local regulatory elements mediated by PaaX, PaaR and PaaY was maintained. Notably, PaaX and PaaR were, for the first time, predicted to co-occur within the same genomic context organization in several groups. In contrast, archaeal genomes predominantly encode incomplete PAA configurations, suggesting functional divergence and/or pathway fragmentation.

Together, these results demonstrate that the PAA pathway is more widespread and evolutionarily dynamic than previously appreciated, combining deep enzymatic conservation with flexible genomic organization. Finally, the conservation of the core epoxidase subunits PaaA and PaaC across PAL-encoding genomes supports their use as robust genomic markers of PAA pathway potential.

**Impact Statement**

By reassessing the phenylacetic acid (PAA) hybrid pathway across thousands of bacterial and archaeal genomes, this study reveals that PAA metabolic potential is far more widespread than previously recognized. Core enzymatic components, including phenylacetate-CoA ligase (PAL) and the PaaA–PaaC epoxidase complex, remain evolutionarily conserved despite extensive genomic and regulatory reorganization. Crucially, we show that conserved motif signatures, integrated with domain architecture and genomic context, provide a reliable basis for high-confidence identification of pathway components, effectively resolving PAL discrimination within the adenylate-forming enzyme superfamily. In addition, our analyses uncover previously unrecognized regulatory configurations associated with PAA pathway organization, highlighting the dynamic interplay between metabolic function and gene regulation. Together, these findings establish a scalable framework for detecting aromatic degradation potential and advance our understanding of how complex metabolic pathways persist and diversify across prokaryotic lineages.

## Data Summary

Genomes are accessible in the Kyoto Encyclopedia of Genes and Genomes database (https://www.genome.jp/kegg/genome/), and hmm Pfam profiles matrices are available in the Interpro database (https://www.ebi.ac.uk/interpro/download/Pfam/). Figures and tables in the supplementary material are available at https://doi.org/10.6084/m9.figshare.31075654 and in the online version of this article.

## Introduction

Aromatic hydrocarbons (AHs) are widespread, occurring as plant-derived compounds, such as lignin, and in fossil sources, like crude oil. They are also found in xenobiotic substances introduced into the environment by anthropogenic activities [1]. Benzene, toluene, ethylbenzene and xylene are particularly toxic and persistent aromatic hydrocarbons that pose a major public health risk. While physical and chemical treatments can reduce their toxicity, complete mineralization is difficult. In contrast, many micro-organisms can fully degrade these compounds by using them as the sole carbon and energy source.

Although some fungi and algae can degrade complex aromatic compounds, extensive evidence shows that bacteria exhibit the greatest metabolic diversity for degrading and mineralizing AHs under both aerobic and anaerobic conditions [2,4]. Under aerobic conditions, aromatic ring activation typically occurs through mono- or dioxygenase-catalysed incorporation of oxygen atoms, increasing molecular reactivity and facilitating subsequent degradation [5]. In contrast, anaerobic activation proceeds via an ATP-dependent reaction catalysed by CoA ligases, forming reactive CoA-thioesters and releasing AMP and pyrophosphate, followed by oxidation and ring cleavage [6]. Benzoyl-CoA and phenylacetyl-CoA (PACoA) are two well-characterized intermediates produced via these anaerobic pathways.

**Fig. 1.**
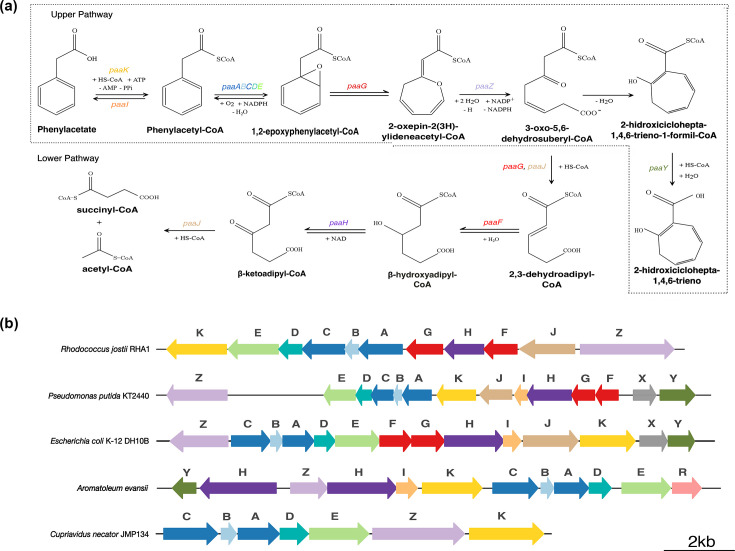
(a) Comparative analysis of gene organization and enzymatic steps of the downstream PAA metabolic pathway in *Rhodococcus jostii* RHA1, *Pseudomonas putida* KT2440, *Escherichia coli* K-12, *Cupriavidus necator* JMP134 and *Aromatoleum evansii*. In the upstream degradation steps, phenylacetate is activated to phenylacetyl-CoA by the ATP-dependent ligase PaaK. The aromatic ring is then epoxidized by the multicomponent monooxygenase complex PaaABCE, yielding a 1,2-epoxide intermediate. This epoxide is isomerized by PaaG to oxepin-CoA, which undergoes ring cleavage and oxidation catalysed by PaaZ to produce 3-oxo-5,6-dehydrosuberyl-CoA, which can spontaneously rearrange to 2-hydroxycyclohepta-1,4,6-triene-1-formyl-CoA, a metabolite known to inhibit PaaZ activity. The thioesterase PaaY may counteract this inhibition by hydrolysing compound 2-hydroxycyclohepta-1,4,6-triene-1-formyl-CoA to 2-hydroxycyclohepta-1,4,6-triene. In the low-level degradation branch, the isomerized ring structure is further processed through a series of reactions involving PaaG, PaaF, PaaH, PaaJ and the bifunctional enzyme PaaZ. These reactions generate intermediates that converge into the *β*-oxidation pathway. (**b**) Genes are colour-coded by function as follows: yellow, phenylacetate-CoA ligase (PaaK); light green, ring epoxidation (PaaE); turquoise, ring epoxidation (PaaD); dark blue, ring epoxidation (PaaA/PaaC); light blue, ring epoxidation (PaaB); red, epoxide isomerization (PaaG/PaaF); purple, 3-hydroxybutyryl-CoA dehydrogenase (PaaH); lilac, oxepin-CoA hydrolase (PaaZ); orange, acyl-CoA thioesterase (PaaI); brown, thiolase (PaaJ); olive green, accessory subunit (PaaY); grey, transcriptional regulator (PaaX); pink, TetR-family transcriptional regulator (PaaR).

These intermediates also participate in a third strategy, the hybrid pathway, which combines features of both aerobic and anaerobic degradation. The phenylacetic acid (PAA) hybrid pathway is an example of this and is commonly divided into an upper (UP) and a lower (LW) pathway (Fig. 1a). The UP pathway is initiated by the ATP-dependent activation of PAA to phenylacetyl-CoA by phenylacetate-CoA ligase (PAL) (encoded by *paaK*), followed by aromatic ring epoxidation by the multicomponent oxygenase complex PaaABC(D)E. The resulting epoxide is subsequently rearranged and hydrolytically cleaved, generating aliphatic intermediates that enter the LW pathway for complete mineralization. Notably, the hydrolytic ring-cleavage step reflects mechanistic features typically associated with anaerobic aromatic metabolism [7,8].

The reaction steps are fully characterized in *Escherichia coli*, with 13 *paa* genes organized into 3 transcriptional units. Two of them, *paaZ* and *paaABCDEFGHIJK*, encode the catabolic genes; the third, *paaXY*, contains the transcription repressor *paaX* [9] (Fig. 1b). In other species, transcriptional regulation is locally modulated by the PaaR repressor protein, a member of the TetR family [9,10], which is typically located within the genomic context organization (GCO) of transcriptional units. Besides *E. coli,* degradation of phenylacetic acid via the hybrid aerobic pathway has also been experimentally characterized in a wide range of bacterial species, including Gram-negative bacteria, such as *Pseudomonas putida* [11], *P. putida* KT2440 [12], *Aromatoleum evansii* [13,14] and *Rhodococcus jostii* RHA1 [15], as well as in Gram-positive bacteria of the genus *Thermus* [16]. Additionally, it has been reported that some Gram-positive bacteria, such as *Clostridium*, produce phenyl acetic acid when growing in a mixture of aromatic amino acids and minerals, which, at a specific pH, can be toxic to the cell, so proposing how these bacteria and other species dispose of this compound deserves investigation [17].

Despite this extensive experimental knowledge, the genomic distribution and evolutionary dynamics of the PAA pathway remain incompletely understood. In the last large-scale bioinformatic survey [8], the authors reported that the *paaA* and *paaC* genes, components of the PACoA epoxidase system, are present in ~16% of 640 complete bacterial genomes, suggesting their potential use as genomic markers. However, predicting an organism’s capacity to fully mineralize PAA into central metabolites such as acetyl-CoA or succinyl-CoA based solely on the presence of individual pathway components remains uncertain.

As in other hybrid and anaerobic AH degradation pathways, the initial activation step is decisive because it commits the substrate to downstream catabolism, exemplified by PAL-mediated activation in the PAA hybrid pathway. Despite its central biochemical role, PAL has rarely been evaluated as a genomic marker in large-scale comparative studies.

PAL belongs to class I of the adenylate-forming enzyme (ANL) superfamily, which is difficult to identify bioinformatically due to the high conservation of the AMP-binding (PF00501) and AMP-binding_C2 (PF14535) domains shared with numerous related ligases/synthetases [18,19]. As a result, even advanced machine-learning and neural network approaches struggle to reliably discriminate among members of the ANL superfamily [20]. Consequently, accurately identifying true orthologs within this family remains a central challenge for functional genome annotation.

Given the relevance of ANL enzymes, such as PAL, in bioremediation, biotechnology and environmental diagnostics, accurately identifying pathway components remains a high priority. The growing volume of genomic data in public repositories offers an unprecedented opportunity to reassess the distribution and diversity of the PAA pathway using comparative genomics tools. Sequence-weighted profiles based on Hidden Markov models, such as those from Pfam [21] and TIGRFAMs [22], have become standard tools for identifying hydrocarbon-degrading enzymes [20,23, 24] and biosynthetic gene clusters, often characterized by conserved GCO patterns [25].

In this study, we aimed to reassess the distribution, organization and evolutionary dynamics of the PAA hybrid degradation pathway by designing and applying a large-scale, integrative bioinformatic framework based on complete, annotated genomes. This framework enabled robust identification of pathway components by integrating protein-architecture profiling, genomic-context organization, motif analysis, phylogenetics and cophylogenetic inference. We applied it to 6,536 fully sequenced bacterial and archaeal genomes to uncover previously unrecognized PAA metabolic potential, reassess the prevalence of complete pathways together with their associated regulators PaaR and PaaX and refine the identification of functional phenylacetate-CoA ligases (PaaK/PAL).

We further evaluated the reliability of the epoxidation subunits PaaA and PaaC as genomic markers of the pathway, characterized gene gain, loss, duplication and rearrangement events shaping pathway architecture, and assessed evidence of horizontal gene transfer, including in archaeal lineages. Finally, through cophylogenetic analyses, we examined whether the coordinated evolution of PaaA–PaaC pairs is associated with pathway configuration and the evolutionary stability of other PAA components across bacterial and archaeal genomes.

## Methods

### Literature inspection

A comprehensive literature search identified organisms expressing PAL and enzymes involved in the hybrid PAA degradation pathway. A posterior genome search was conducted in the Kyoto Encyclopedia of Genes and Genomes (KEGG) [26,27] and GenBank databases [28] to update genomic and metabolic features, such as gene names and sequences of both genes and proteins for the 13 proteins in the pathway and for the 2 local transcriptional regulators (TFs) (Table S1, available in the online Supplementary Material).

### Construction of protein architectures of the PAA degradation pathway

We identified the protein architectures of each enzyme in the PAA pathway using a methodology previously developed by our research group [23,29, 30]. This approach uses the latest version of Pfam-A matrices [21] and hmmscan [31] to detect non-overlapping domain combinations that cover the maximum length of each amino acid sequence. In a first step, domains with an *e*-value <0.001 were selected. Then, because some of these domains overlap, we chose those with the lowest expectation value spanning the most extended possible sequence length, which was defined as the representative architecture within each protein. This approach enables a conserved architectural definition, which is particularly helpful when proteins are not assigned to KEGG ortholog groups [27] or Cluster of Orthologous Groups [32].

### Identification of protein architectures in complete proteomes

We downloaded a set of proteomes from the KEGG database [33] (December 2022 release, purchased and locally curated by our group), initially comprising 7,030 genomes. After excluding 695 eukaryotic genomes, as well as partial assemblies and low-quality prokaryotic genomes, a total of 6,536 fully sequenced and annotated prokaryotic genomes were retained for downstream analyses. These genomes span both prokaryotic domains (Bacteria and Archaea), including 380 archaeal genomes (Kanehisa and Goto, s. f.). Furthermore, strain-level genomes were deliberately retained, as our results and previous studies of our group [23,29, 30] indicate that pathway remodelling can occur at this level, and restricting the analysis to representative genomes would mask this variation. We also downloaded the proteome of *A. evansii* (SRR27236985) from the SRA database [34], a bacterium in which the PAA pathway has been extensively studied [35] and is absent from the KEGG compendia.

To identify homologous sequences, we conducted an ‘hmmrscan’ search from the HMMER suite [31] against our proteome database using the Pfam-A database [21], to identify the protein architectures defining each protein and the TFs describing the PAA pathway. To maximize sensitivity, the scan was performed using the parameters -E 0.001, -domE 0.1 and -max, which enabled full-sensitivity mode and bypassed heuristic filters. Hits with *e*-values ≤0.001 were considered significant.

### Genomic context inspections

PAA enzymes are typically organized into clusters that share the genomic neighbourhood. It is also reported that the PAL is an enzyme catalysing the first reaction in the hybrid PAA degradation pathway. To inspect the GCO, we selected PAL homologous sequences containing conserved hits for AMP-binding and AMP-binding_C_2, where AMP-binding at the N-terminus and AMP-binding_C_2 at the C-terminus were preserved [18].

We limited the GCO inspection to 20 genes upstream and 20 genes downstream of the *paak* gene (PAL), since, as seen in Fig. 1b, the organization displayed by the experimentally identified organisms showed at least 14 proteins in the GCO, including the epoxidases, isomerases, hydrolases, thioesterases, hydratases, dehydrogenases and the local TFs (PaaX and PaaR). However, in some cases, we observed genes or pseudogenes that disrupted the experimentally proposed cluster models of the pathway; therefore, inspecting a window of 20 flanking genes was adopted as a reasonable threshold for genomic context analysis. When pseudogenes or disrupted regions were detected, we performed blastx searches [36] to validate gene annotations (Table S2). Positive hits were subsequently examined for the presence of the expected protein domains, and when confirmed, the corresponding enzyme or transcription factor was incorporated into the reconstructed pathway (Table S2). This scope reflects the commonly observed operon organization, which typically includes at least nine enzymes associated with PAL [9,15, 37].

In the first round, we retained all hits that included at least one of the architectures describing an enzyme in the pathway context. In a second round, we separated the PALs and their contexts, preserving the multicomponent PaaA and PaaC enzymes, which shared the same protein architecture (PaaA_PaaC), as these enzymes were classified as genomic markers. Additionally, some genomes contained multiple copies of the PAL enzyme, sometimes lacking the PaaA and PaaC multicomponent proteins. These sequences and their contexts, along with other enzymes observed in the experimental models, were also preserved to trace their possible origin.

### Identification of PAL motifs

To identify conserved motifs in PAL sequences, we used the MEME suite [38] on a dataset of 332 non-redundant PAL homologs. These non-redundant sequences were selected using CD-HIT with default parameters [39]. When CD-HIT failed to retain an experimentally characterized PAL within a cluster, we manually replaced the representative sequence with one supported by experimental evidence (Table S3). MEME was configured to search for at least 10 motifs, with lengths ranging from 6 to 30 amino acids. These parameters were chosen based on previously reported motifs in class I, subclass Ib aryl-CoA ligases [18,40]. The resulting motif matrices were used to scan PAL homologs with the MAST program [41] for conserved protein domains (AMP-binding and AMP-binding_C2). We filtered the result hit by the corresponding GCO.

### Phylogenetic reconstruction

Sequence alignment was performed with muscle 5 [42], and the alignment was trimmed with default parameters using trimAl [43]. Phylogenetic analysis was conducted using IQ-TREE (multicore version 1.6.12 for 64-bit Linux), employing maximum likelihood inference with the approximate likelihood-ratio test and 1,000 bootstrap replicates to determine the optimal substitution model [44]. The chosen model was LG+R10 according to BIC. Two representative benzoate-CoA ligase (BCL) sequences from [23] were included as outgroup references. The final tree, comprising 855 sequences, was visualized using the Interactive Tree of Life (iTOL) tool [45].

The same procedure was applied to calculate the PaaA and PaaC tree topology. The LG+F+R8 model was chosen for both trees based on BIC.

### Structural PAL comparison

Structural similarity between the Alphafold-3 [46] of 13 predicted proteins, representing all taxonomic classes and paralogs, and the reference crystallographic structure 2Y4N (chain B) was evaluated using a complementary framework integrating both local and global structural metrics. First, Cα root mean square deviation (RMSD) values were calculated following optimal structural superposition using the *super* algorithm implemented in PyMOL [47], with the analysis restricted to protein residues. For each comparison, both the RMSD and the number of Cα atoms included in the alignment were recorded, enabling estimation of the size of the conserved structural core. This procedure applies iterative refinement to minimize the influence of divergent regions or structural outliers. In parallel, global fold similarity was quantified using the TM-score, computed with the TM-score program (Zhang and Skolnick) [48], which provides a length-normalized measure sensitive to overall architectural similarity.

### Analysis of co-evolving pathway gene markers

To assess phylogenetic congruence between PAL and PaaA-C genomic markers, we first computed the normalized Robinson–Foulds distance using *ape* [49] and *phangorn* [50]. We then evaluated cophylogenetic signal using the Procrustean Approach to Cophylogeny (PACo) [51]. Patristic (cophenetic) distance matrices were calculated with *cophenetic* [52] for PaaA-PaaC. An identity association matrix was linked to each pair tip to its corresponding. The matrices were combined with *prepare_paco_data*() (paco), and Cailliez’s correction was applied (*add_pcoord*, correction = ‘cailliez’) to ensure Euclidean embeddability before Procrustes superimposition. PACo was run with 1,000 permutations (nperm=1,000; seed=42; method = ‘r2’). Statistical significance was assessed by permutation testing, with low *P*-values indicating overall phylogenetic congruence.

### Residual-based link classification

Interaction residuals were extracted using *residuals_paco*() to quantify local fit for each marker pair. Low residuals indicate putative codivergence, whereas high residuals suggest incongruence that may result from events such as paralogy, recombination, host switching or rate heterogeneity. Residuals were classified using dataset-specific quantiles: low (<Q1≈1.40), medium (Q1–Q3≈1.41–2.40), upper-medium (2.42–3.39) and high (≥p90≈3.45). Extreme outliers were defined as residuals >Q3+3×IQR (≈5.42). Results were visualized with *ggplot2* and as mirror trees (tanglegrams) in iTOL [45].

### Correlations between pathway configuration and co-evolutionary pairs

Associations between configuration and residual class were analysed using categorical association methods. The overall effect size was quantified using bias-corrected Cramér’s V in the ‘DescTools’ R library [53]. Statistical significance was evaluated using both a Pearson *χ*² test and a permutation-based general independence test (9,999 resamples) to account for sparse and uneven contingency tables, using the ‘coin’ library. Standardized *χ*² residuals were calculated to identify configuration–residual combinations driving departures from independence. Plots were generated using the ‘pheatmap’ function from the R package [54].

## Results and discussion

### Identification of PAL probable orthologues and closely related paralogs

Using PAL protein architecture recognition, we identified 12,240 homologous sequences across 4,375 genomes. Motif scanning for conserved catalytic and substrate-recognition residues reduced this set to 2,161 candidates (Table S4). A final genomic-context filter retained only sequences that co-localized with the PaaABCDE epoxidase complex and with genes from the experimentally validated upper and lower segments of the PAA pathway.

### Description of conserved PAL by motifs

**Fig. 2.**
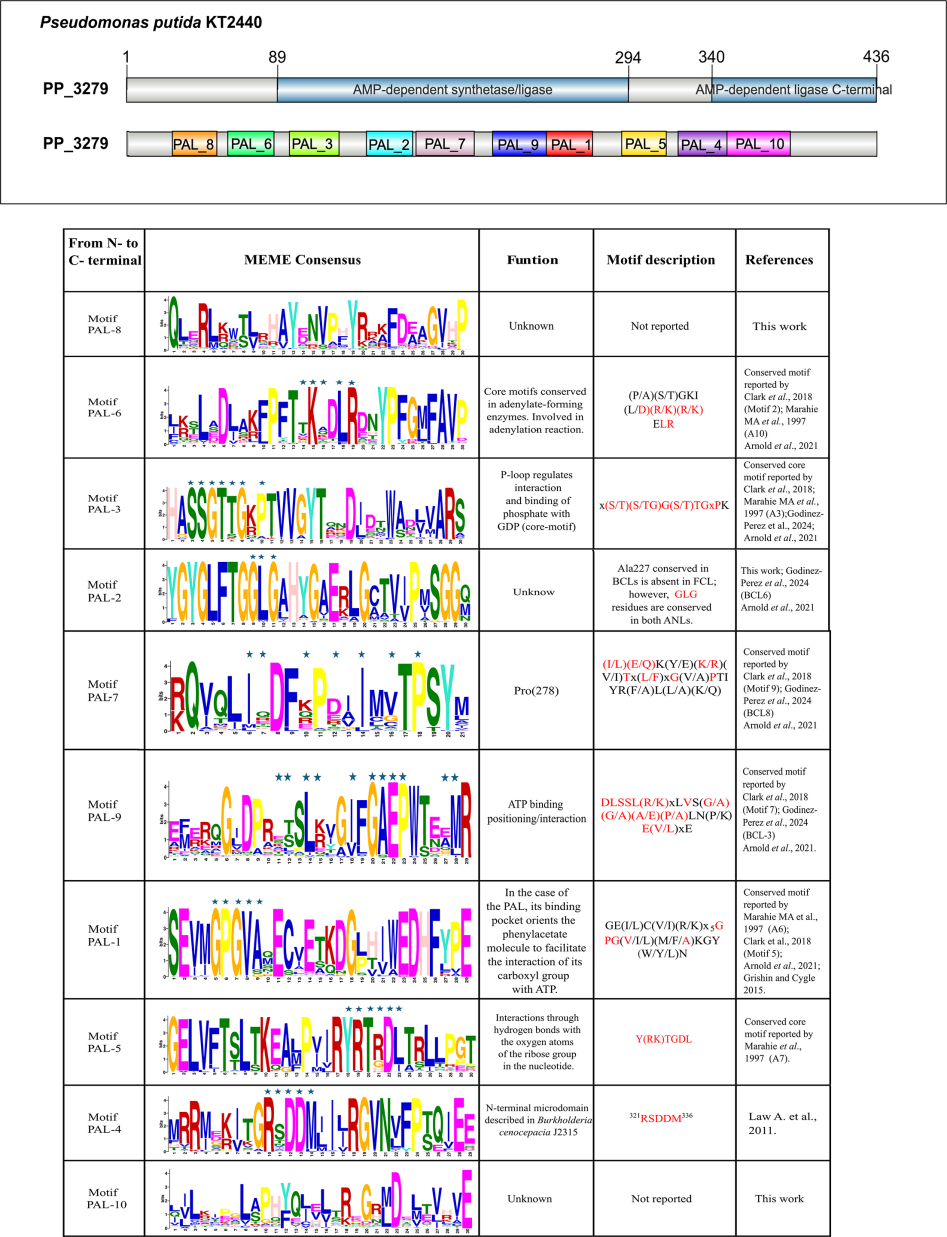
Identification and organization of conserved sequence motifs in PAL enzymes. Top panel: domain architecture of the PAL protein PP_3279 from *Pseudomonas putida* KT2440, showing the N-terminal AMP-dependent synthetase/ligase domain and the C-terminal AMP-dependent ligase region, with the relative positions of the ten PAL motifs (PAL-1 to PAL-10) indicated along the sequence. Bottom panel: summary of PAL motifs identified by MEME analysis, ordered from the N- to the C-terminus.

We identified a group of core motifs common in the ANL enzyme superfamily [18,23, 40, 55, 56]. Among these, a key motif in the N-terminal region, PAL-3, includes the conserved SSGTGG sequence (Fig. 2). This motif is associated with the phosphate-binding loop (P-loop), as described by [18]. Another core motif, PAL-1, located in the N-terminal region, plays a crucial role in substrate recognition. In PAL enzymes, this motif is located within the binding pocket, where it helps orient the phenylacetate molecule, enabling its carboxyl group to interact with ATP [57]. PAL-5, another recognized core motif, contains the conserved sequence ‘Y(RK)TGDL’, which forms hydrogen bonds with the oxygen atoms of the nucleotide’s ribose group [56]. The conserved ‘RSDDM’ sequence in PAL-4 has been identified as part of a microdomain in the N-terminal region of *Burkholderia cenocepacia* J2315I [55]. Motif PAL-2 includes the conserved ‘GLG’ sequence, shared by both PAL and BCL. This motif is located two residues downstream of Ala227 in BCL ligases of *Rhodopseudomonas palustris* and is also conserved among BCLs of *Betaproteobacteria* and *Alphaproteobacteria* [23]. However, this alanine is replaced by a conserved threonine in the PAL sequences analysed in this work (Fig. S1). Additionally, we report two motifs not previously identified in other ANL enzymes (Fig. 2), for which we were unable to assign a probable function. Together, these results indicate that, despite the overall conservation of the ANL structural framework, PAL enzymes can be distinguished by a specific combination of conserved and lineage-specific motifs. This motif architecture provides a robust basis for discriminating PALs from closely related ANL enzymes, overcoming a major limitation in the functional annotation of this superfamily. Importantly, integrating motif signatures with genomic context analysis enhances the specificity of PAL identification, particularly for large-scale, fragmented datasets, such as metagenomes and metagenome-assembled genomes (MAGs).

### Genomic context selection of PAL

Analysis of GCO showed that the protein architectures encoded by paaA, paaB and paaC, as well as the transcriptional repressor paaX, are rarely found outside the PAL genomic context, whereas homologues of PaaG**,** PaaF, PaaI, PaaJ, PaaY and PaaZ are frequently conserved out of the GCO (Fig. S2). This contrast likely reflects differences in domain specificity: PaaA–PaaC encode domains highly specialized for phenylacetate metabolism, while other components contain more broadly distributed domains. For instance, PaaD includes the FeS_assembly_P domain (PF01883), involved in diverse cellular processes [58], and PaaE carries FAD_binding_6 and NAD_binding_1 domains (PF00970) found in multiple oxidoreductases [59], explaining their presence outside the PAL GCO.

**Fig. 3.**
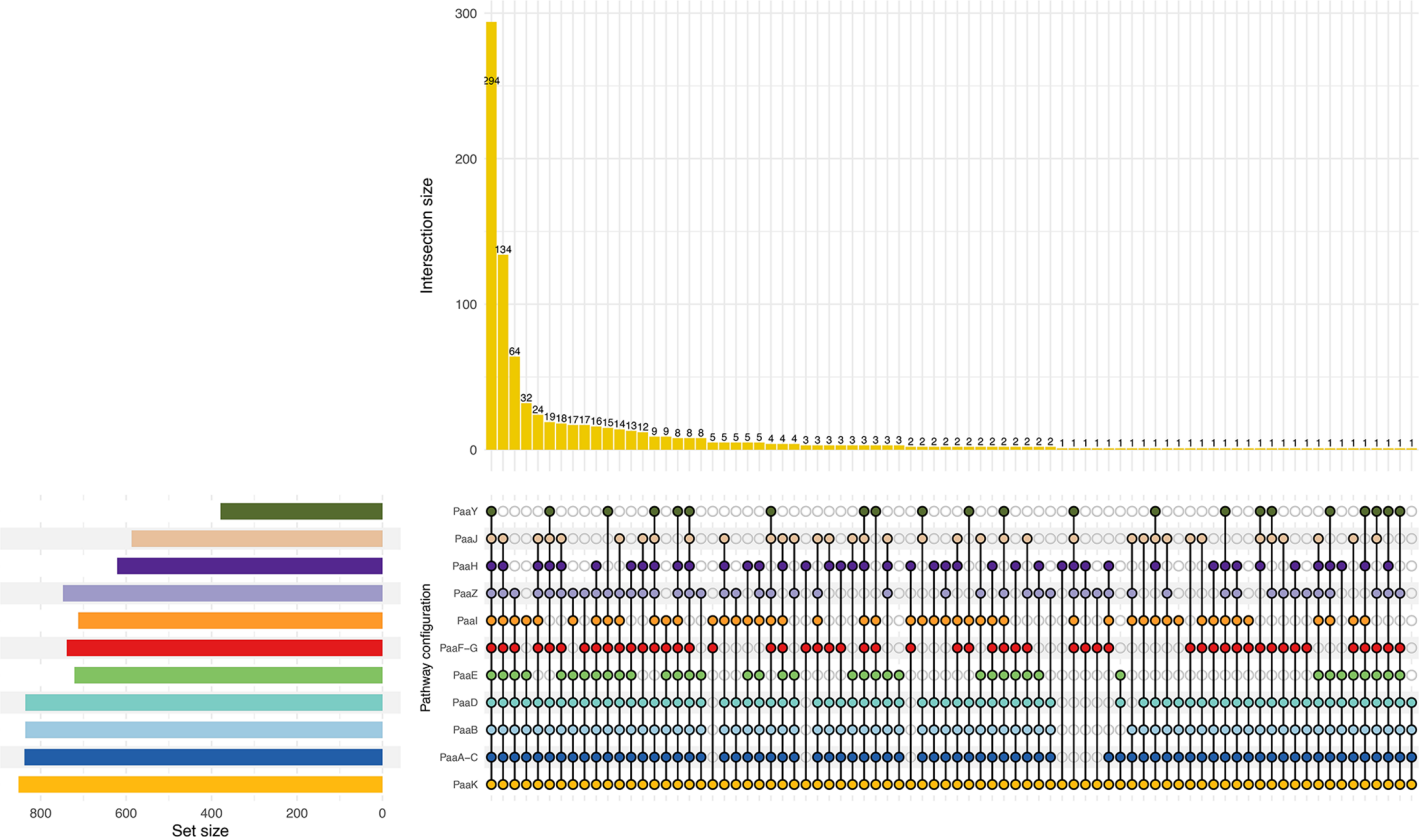
Co-occurrence patterns of PAA pathway components across PAL-containing genomes. The UpSet plot summarizes the intersection sizes of PAA pathway genes (left matrix), highlighting the frequency with which individual enzymes co-occur across genomic contexts. Bars indicate the number of genomes sharing a given combination of pathway components, while set sizes reflect the overall prevalence of each gene. The core epoxidation module (PaaA–PaaC) shows the highest and most consistent co-occurrence, whereas other components display variable presence, illustrating extensive pathway remodelling across taxa.

Within the PAL GCO, *paaA*, *paaB* and *paaC* show strong positional conservation (Fig. 3), consistent with their essential role in aromatic ring opening during phenylacetate degradation. Although PaaD and PaaE may contribute to this process, experimental studies indicate that they are non-essential under certain conditions and strains [8,57]. Structurally, the catalytic subunit PaaA and the structural subunit PaaC are connected by PaaB, reinforcing the functional cohesion of this core complex [60].

A previous computational study proposed PaaA and PaaC as genomic markers based on their conservation in ~16% of surveyed bacterial genomes [8]. Accordingly, genomes harbouring *paaA* and *paaC* in the PAL context are more likely to encode orthologs of experimentally characterized PAA enzymes. Applying this criterion, we identified 845 PALs. Notably, nine *Cupriavidus* proteomes contain paralogous PAL copies that lack a complete epoxidation module (EM) in the PAL GCO, while retaining components of the upper or lower pathway (Table S5). PAL duplication has been experimentally documented in *B. cenocepacia* J2315 [55] and in *Cupriavidus* species. In *B. cenocepacia* J2315, the chromosomal copy (reported as PaaK1) exhibits lower affinity for phenylacetate than the copy encoded on the secondary chromosome (reported as PaaK2). Comparable differences have been reported for BCLs in *B. cenocepacia* LB400, where the chromosomal enzyme shows lower affinity than the chromid-encoded copy [61].

Paralogous redundancy among aryl-CoA ligases is widely documented [62,63] and is thought to enhance fitness under fluctuating environmental conditions and variable oxygen availability. In addition, the uneven distribution of pathway enzymes across replicons, such as BCL, suggests selective retention of megaplasmid-encoded copies when chromosomal components are lost [23]. This model is supported by differences in substrate affinity, replicon localization, transcriptional organization and recurrent chromosomal gene loss [23] as well as experimentally demonstrated variation in BCL substrate specificity [55]. Together, these findings indicate that ACL redundancy, including PAL, promotes metabolic flexibility and adaptation in environments rich in monoaromatic compounds.

### Structural comparison of predicted PALs

Structural comparison of the ten PAL orthologs and paralogues against *B. cenocepacia* J2315 [55,60]. Structural superposition using PyMOL revealed strong conservation of the structural core across most predicted models relative to the reference crystallographic structure (Fig. S3) with RMSD(Cα) values ranging from 0.36 to 1.35 Å (Table S6). However, the proportion of Cα atoms contributing to each alignment varied substantially, ranging from ~60–95% of the residues in the crystal structure. Models with extensive alignment coverage (≈90–95%) also displayed high TM-scores (>0.8), consistent with robust conservation of both the structural core and the global fold. In contrast, several models exhibited low RMSD values despite moderate to low TM-scores (<0.5) (Table S6), indicating that structural similarity in these cases is largely confined to a conserved core, whereas peripheral regions, insertions or non-equivalent domains introduce marked divergence in overall architecture. Collectively, these results indicate that the predicted structures faithfully recapitulate the experimentally defined structural core, whereas the degree of complete fold conservation varies among models, largely due to differences in peripheral or accessory regions. This is exemplified by the paralog bceo-I35_5581, belonging to *B. cenocepacia* H111 (Fig. S4), annotated in the KEGG database as an enoyl-CoA hydratase/isomerase, which, in our predictions, includes the characteristic AMP-binding domains and an additional ECH_1 domain, consistent with the domain architecture described for PaaF, revealing the structural plasticity that likely underpins functional diversification within the PAA pathway.

### Taxonomic distribution of the PAA pathway

Including probable orthologs and paralogs, we predicted PAL and downstream PAA pathway enzymes in 853 bacterial and archaeal proteomes. Members of the phylum *Pseudomonadota* dominated the dataset, accounting for 66% of the bacterial sequences (821 proteomes), and included representatives of the *Gamma*-, *Beta*- and *Alphaproteobacteria*. Then followed by *Actinomycetes* (21%), *Bacilli* (8.8%) and *Flavobacteria* (1.6%). Minor contributions (2.19%) came from *Clostridia*, *Cytophagia*, *Deinococci*, *Hydrogenophilia*, *Thermoleophilia* and *Thermomicrobia*, the latter three encoding only PAL. In addition, 32 PAL-associated pathways were identified in archaeal proteomes of the *Halobacteria* class (Fig. S5).

Taxonomic distribution revealed extensive diversity in PAA pathway organization, reflecting both functional and evolutionary variability across the 834 micro-organisms analysed. To interpret this diversity, we adopted the conventional subdivision of the pathway into upper and lower levels [57] and further classified genomic contexts based on regulatory content. Specifically, we recorded the presence of the local transcriptional regulators PaaR and PaaX and the metabolic modulator PaaY within the GCO. Based on gene content, GCOs were categorized as full pathway (FP), UP, EM [PaaABC(D)E], ‘partial multicomponent monooxygenase’ (PaaA–PaaC only) or ‘other configurations’ lacking core epoxidase subunits in paralogs (Table S7).

**Fig. 4.**
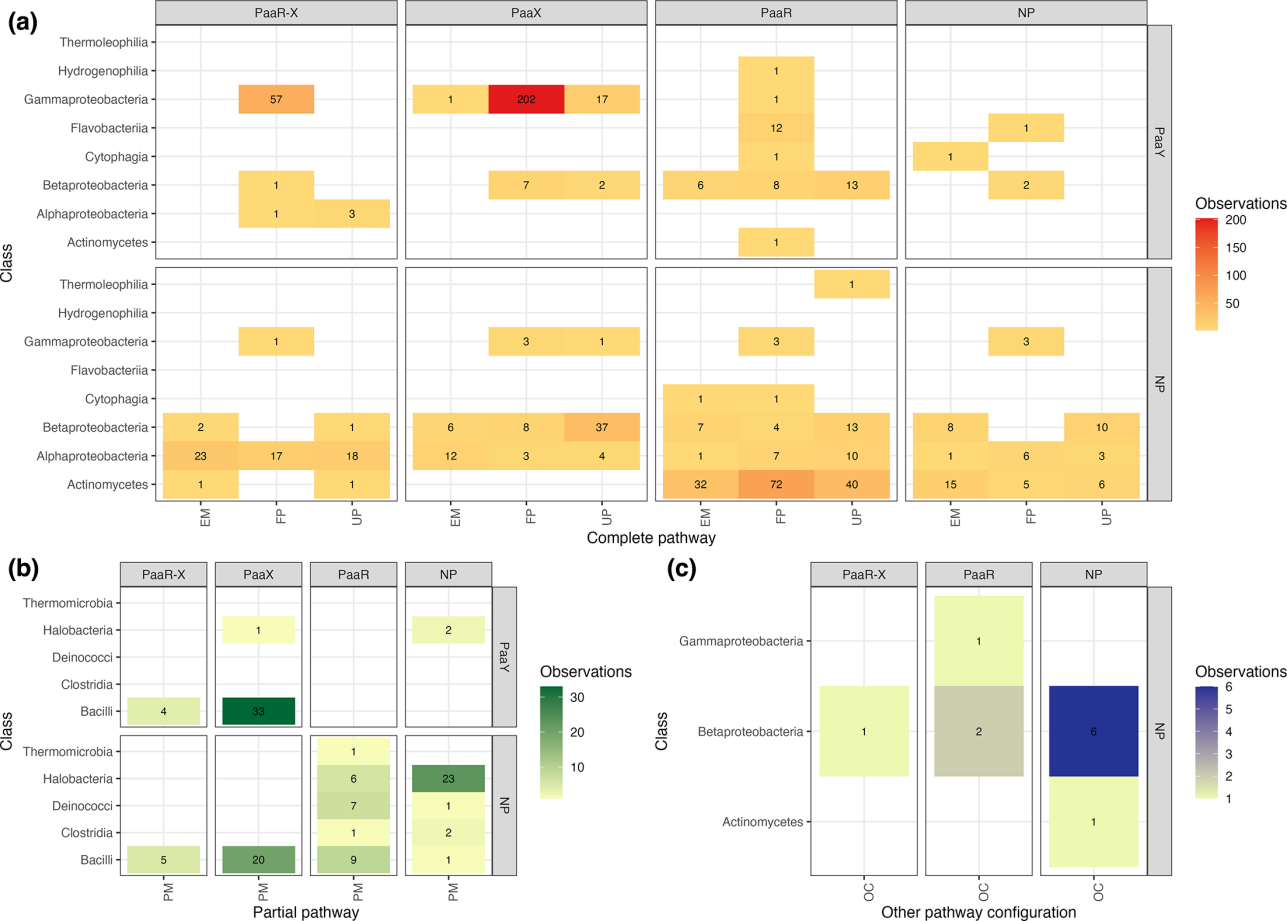
Distribution of PAA pathway configurations and regulatory architectures across PAL-containing genomes. (**a**) Heatmap showing the co-occurrence of complete pathway configurations (EM, FP, UP) with local transcriptional regulators [PaaR–X, PaaX, PaaR or no regulator (NP)] across major bacterial classes. Cell values indicate the number of genomes exhibiting each combination, with colour intensity reflecting the number of observations. (**b**) Distribution of partial multicomponent (PM) configurations across bacterial and archaeal classes, highlighting the prevalence of specific regulatory states associated with reduced epoxidation modules. (**c**) Occurrence of other pathway configurations (OC) lacking the canonical epoxidation module, illustrating rare and lineage-specific arrangements.

Gammaproteobacteria showed the highest representation of FP configurations, commonly regulated by PaaX alone or in combination with PaaR, and frequently encoding PaaY (Fig. 4a). Loss of PaaY was rare in this class and occurred across multiple regulatory backgrounds. Additional UP, EM and OC configurations were also observed, including a *Pseudomonas eucalypticola* paralog lacking most epoxidase components and classified as OC (Fig. 4c).

In *Betaproteobacteria*, FP, UP and EM configurations were detected across diverse regulatory arrangements. Although 66% of species lacked PaaY, PaaX and/or PaaR, these genes were frequently retained, particularly in UP configurations. PM configurations were absent, while a small number of OC arrangements lacked both PaaY and local transcription factors (Fig. 4b, c).

*Alphaproteobacteria* and *Actinomycetes* displayed a marked scarcity of PaaY, with only isolated exceptions. In *Alphaproteobacteria*, a single FP encoding PaaY–PaaR–PaaX was identified in *Gemmobacter fulvus* con5 [64]. Most representatives exhibited FP, UP or EM configurations regulated by PaaR, PaaX, both or neither (Fig. 4a). Actinomycetes showed the highest frequency of PaaR predictions, predominantly in FP, UP and EM contexts lacking PaaY. Only one *Actinomycetes*, *Corynebacterium cystitidis* NCTC11863, encoded PaaY together with PaaR [65]. At the same time, rare PaaR–X configurations were observed in *Prauserella marina* DSM 45268 [66] and *Mycobacteroides abscessus* ATCC 19977 [67].

Complete pathways were uncommon in *Hydrogenophilia*, *Cytophagia*, *Flavobacteriia*, *Thermomicrobia* and *Thermoleophilia*, representing novel observations for several of these classes. *Flavobacteria* were notable for a strong association between FP configurations and PaaY–PaaR. In contrast, the only representatives of *Hydrogenophilia*, *Thermoleophilia* and *Thermomicrobia* exhibited FP, UP and a partial multicomponent configuration, respectively.

Within *Bacillota*, PAL predictions were almost exclusively associated with PM configurations. In *Bacilli*, approximately half of the genomes encoded PaaY together with PaaX, while the remainder lacked PaaY and showed variable retention of PaaX and/or PaaR. One soil isolate, *Aneurinibacillus soli* CB4, lacked both regulators and PaaY. The few *Clostridia* analysed also displayed PM configurations, including *Sulfobacillus thermotolerans* Kr1, which encoded PaaR but lacked PaaY.

In contrast to bacterial PAL-associated pathways, archaeal PAL-associated pathways were rare and highly constrained. All 32 archaeal cases, confined to *Halobacteria*, exhibited a PM configuration. Only three encoded PaaY, and among these, *Haloterrigena turkmenica* DSM 5511, isolated from sulfate-saline soil [68], also carried a local PaaX regulator. Most *Halobacteria* lacked both PaaY and local transcriptional regulators, underscoring either limited regulatory diversification of the PAA pathway in Archaea or a reliance on syntrophic environmental associations common in anaerobic metabolisms [69,70].

### Origin and remodelling of the PAA pathway

The PAA hybrid pathway is distributed across multiple taxonomic groups. However, as previously noted, the 13 enzymes involved, along with their associated metabolic and transcriptional regulators, are not fully conserved and do not preserve synteny in experimentally validated pathways. Our results support this observation among the 834 microorganisms, in which we identified diverse configurations that reflect both functional and evolutionary variability.

**Fig. 5.**
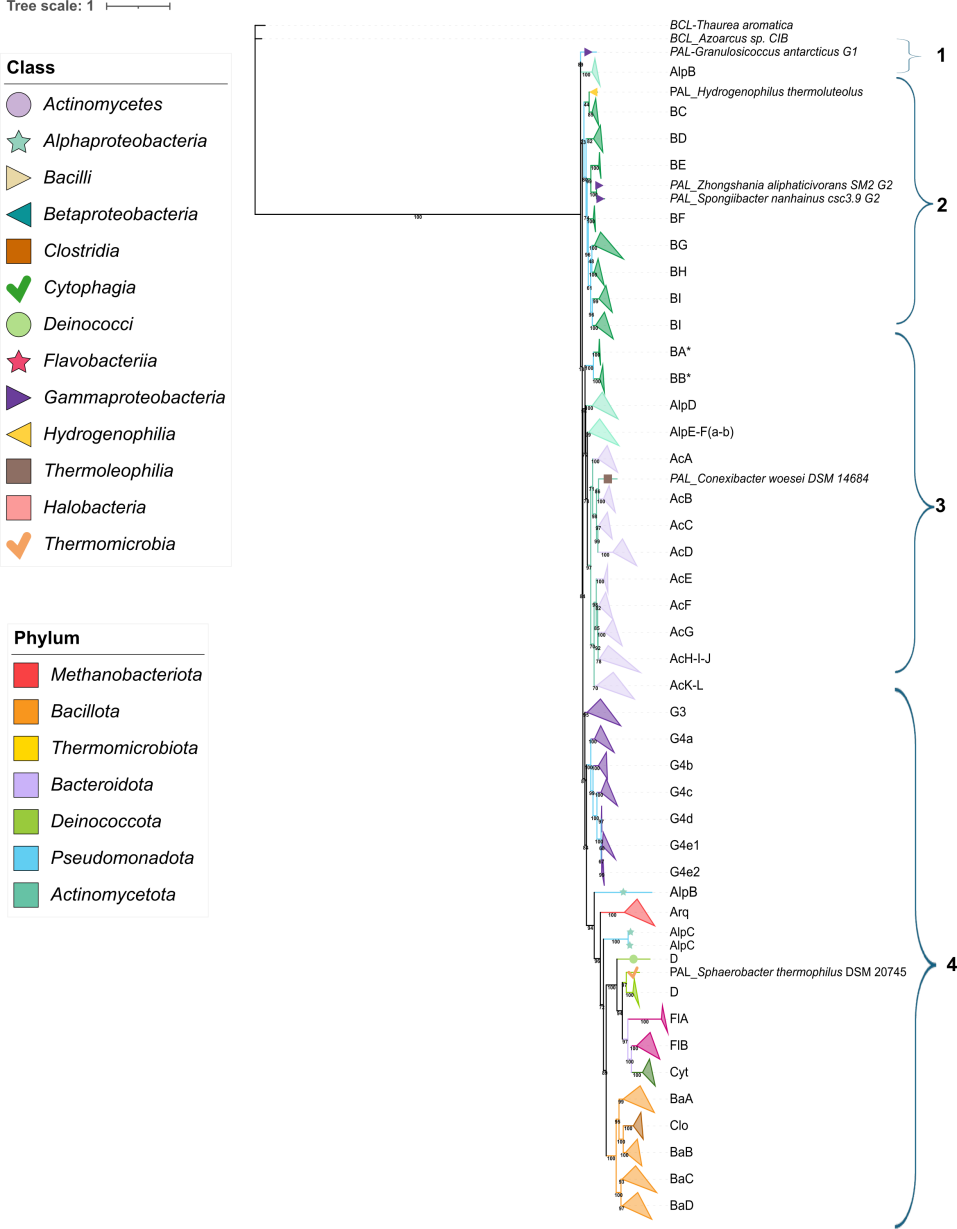
Phylogenetic reconstruction and clade-level organization of PALs. Maximum-likelihood phylogeny inferred from representative PAL sequences, rooted with related aryl-CoA ligases (BCLs). Major monophyletic groups are indicated by brackets (clades 1–4) and subdivided into named subclades. Tip symbols and colours denote taxonomic class, while background shading indicates phylum affiliation. Bootstrap support values are shown at key nodes, and branch lengths are proportional to sequence divergence.

To investigate the evolutionary history of genomic rearrangements in this pathway, we constructed a rooted phylogenetic tree from 853 PAL sequences. We associated them with their corresponding phyletic profiles (Fig. S6). The sequences clustered into four major monophyletic groups (Fig. 5): three groups containing PALs predicted in *Pseudomonadota* and a fourth composed of PALs from non-*Pseudomonadota* genera, diverging from *Gammaproteobacteria*.

To propose a model for PAA pathway remodelling based on the 853 PAL sequences, we constructed a reduced PAL phylogeny comprising representative sequences from each subclade. This tree highlights the distinct pathway configurations and replicon organizations identified among the sequences, together with their distribution across genera (Table S8). Importantly, the reduced subtree, reconstructed from 186 representative PAL sequences, retained the overall topology observed in the phylogeny inferred from the complete set of 853 PAL predictions (Fig. S7).

## Clade 1

### Early gammaproteobacterial origin and initial pathway remodelling

Root-to-tip analysis suggests that the PAA pathway originated within *Gammaproteobacteria*, represented by *Granulosicoccus antarcticus* IMCC3135, which encodes an FP configuration regulated by PaaR but lacking PaaY. Although functional, this architecture may be less efficient due to the potential accumulation of 1,2-epoxyphenylacetyl-CoA, a toxic intermediate [71], a deficiency that is partly mitigated by the presence of lower-pathway enzymes and PaaI.

Within the same clade, the first remodelling event appears in a small alphaproteobacterial group (AlpA), comprising six *Azospirillum* species. One divergent lineage shows loss of lower-pathway genes and the transcriptional regulator, yielding a UP system encoded on a secondary chromosome. Subsequent re-acquisitions and losses of FP configurations occur within *Azospirillum*, with no detectable PaaR/PaaX homologs in the GCO.

## Clade 2

### *Betaproteobacteria* as the primary hotspot of PAL diversification

*Betaproteobacteria* (BC–BJ) form the most diverse region of the phylogeny. Early branching subclade BC, including *Hydrogenophilus* and *Rhodocyclales*, retains FP-PaaR and shows the earliest occurrence of PaaY, followed by progressive losses of PaaY and lower-pathway genes in *Azoarcus* and *Thauera. Aromatoleum* displays additional remodelling, with FP and UP systems coexisting, and the EM configuration occurring only in paralogs on distinct replicons (subclade BE) (Table S8). Similar duplication patterns have been described for BCLs [23,62] and are likely to promote metabolic flexibility under variable environmental conditions.

In *A. evansii*, we detected a complete PaaABCDE complex with accessory genes (*paaZ*, *paaI*, *paaH* and *paaY*), although *paaG*, *paaF* and *paaJ* were absent, differing from previous descriptions [14,35], perhaps reflecting updates or inconsistencies in the two available genome assemblies – one produced with PacBio and the other with Illumina technologies.

Subclade G2 contains two *Gammaproteobacteria* (*Zhongshania*, *Spongiibacter*) clustering with *Rhodocyclales*, consistent with possible recent horizontal acquisition. *Zhongshania* encodes the first FP–PaaY–PaaX configuration, whereas *Spongiibacter* uniquely retains both PaaR and PaaX in the GCO. The coexistence of both regulators within the same strain has not been previously reported; however, our analysis revealed their presence in strains of Pseudomonadota and in several *Bacilli* (Fig. S7).

Further transitions appear in multiple subclades. In the case of BF, the strains in the genus *Herbaspirillum* showed a FP–PaaY–PaaX and UP–PaaR configurations. *Herbaspirillum* is known for its ability to degrade hydrocarbons (Martín, s. f. [62,72]; .

Subclade BG, which clusters strains belonging to *Janthinobacterium* and *Telluria*, showed a clear UP-to-FP transition, with variable reacquisition of PaaY and conservation of PaaX as a TF in the genomic context

Subclade BH clustered a group of *Burkholderiales*, presenting the broadest spectrum of architectures, including chromosomal EM systems and FP/UP variants located on secondary replicons or plasmids (Fig. S7 and Table S5). Several species encode PAL paralogs located in the BB subclade associated with an OC context. The terminal BI–BJ subclades include ecologically diverse genera and exhibit the full spectrum of pathway and regulatory configurations, highlighting. Together, these patterns confirm that *Betaproteobacteria* are the principal centre of innovation and genomic reorganization in the PAA pathway.

## Clade 3

### *Alphaproteobacteria* conserved FP systems contrasted with extensive marine remodelling

A small set of *Alphaproteobacteria* and several betaproteobacterial paralogs form subclade BA, which includes *B. cenocepacia* J2315, whose PaaK paralogs have been structurally and mechanistically characterized [55]. Subclade BB contains *Cupriavidus* strains with OC-type architectures lacking the PaaABCDE complex; OC-type copies occur on the main chromosome, with paralogs located on secondary replicons (Table S5). These patterns mirror known functional redundancy in *Burkholderiales* [23,62, 73], in which coordinated expression across replicons appears essential for aromatic compound degradation.

Subclade AlpD includes *Sphingosinicella*, *Sphingomonas* and *Novosphingobium*. The only predicted PAL in *Novosphingobium pentaromativorans* US6-1 is plasmid-encoded (pLA3), as is a PAH-degrading cluster typically residing on plasmids in this genus [74].

The remaining alphaproteobacterial PALs fall into the subclades AlpE and AlpF, which share a last common ancestor. Subclade AlpE spans diverse genera, including *Acetobacter*, *Bartonella*, *Bosea*, *Bradyrhizobium*, *Methylobacterium*, *Microvirga*, *Pseudorhodoplanes*, *Rhodopseudomonas*, *Roseomonas*, *Tardiphaga*, *Variibacter* and *Xanthobacter* and exhibits multiple FP, UP and EM configurations. Gains and losses of pathway enzymes and regulators are common; *R. palustris* contains two PAL copies (UP and EM), both PaaR-regulated. However, *R. palustris* is recognized as a hydrocarbon degrader and encodes additional ACLs, including a BCL; the PAA pathway has not yet been experimentally characterized in this species.

Subclade AlpF shows a non-monophyletic structure, defined by a series of successive branching events rather than a single, well-supported basal node. This subclade contains PALs from *Rhizobiales*, including *Rhizobium/Agrobacterium*, *Ensifer/Sinorhizobium*, *Mesorhizobium* and *Bradyrhizobium*, which predominantly encode FP configurations accompanied by PaaX or PaaR–X on chromosomes, chromids or megaplasmids. PaaY is rare, observed only in a few strains of *Gemmobacter* and *Rhizobium/Agrobacterium*, as in the only representative of *Paroceanicella*, which retains PaaY in an UP–PaaR-X configuration.

In contrast, marine *Alphaproteobacteria,* such as *Roseobacter*, *Labrenzia*, *Dinoroseobacter* and *Sulfitobacter,* display extensive pathway fragmentation (FP/UP/EM), heterogeneous regulators and frequent loss of PaaY. Overall, *Rhizobiales* preserve a more ancestral FP-PaaY module, whereas marine lineages exhibit pronounced diversification.

### *Actinomycetes* exhibit a three-lineage radiation, highlighting the reduction and innovation of the PAA pathway

This clade represents a distinct radiation of actinobacterial PALs that can be broadly divided into three groups. The first comprises the monophyletic subclades AcA–AcD. AcA includes PALs from *Actinoplanes*, *Aeromicrobium* and *Nocardioides*, all of which retain PaaR in the GCO but show progressive pathway reduction. AcB contains strains belonging to the genus *Mycobacterium*, *Mycolicibacterium* and the *Thermoleophilia Conexibacter woesei*, all of which have UP configurations regulated by PaaR. A similar UP pattern is observed in *Gordonia*, *Nocardia*, *Rhodococcus* and *Tsukamurella* (AcC), with occasional loss of PaaR and a single acquisition of PaaX (*M. abscessus*). In AcD, most *Corynebacterium* strains gain an FP configuration while retaining PaaR [75], as previously reported, and one strain exhibits the first PaaY-regulated FP system reported for this genus.

The second group (AcE–AcG) includes *Nocardiopsis*, *Actinopolyspora*, *Saccharopolyspora* and *Streptomyces*. These lineages encode FP or EM architectures, in which PaaR appears to be conserved in the GCO, whereas EM is ubiquitous in *Streptomyces*. The third group (AcI–AcL) encompasses diverse genera, such as *Agromyces*, *Arthrobacter*, *Leucobacter*, *Microterricola* and *Rothia*, in which FP and UP configurations coexist, and PaaR again predominates. However, PaaX and TF-absent states occur sporadically. Overall, the TF PaaR is the dominant TF, the metabolic PaaY regulator is rare and nearly all PAL genes are chromosomal, consistent with vertical inheritance and stable, low-plasmid genomes.

These presences of the PAA pathway in members of *Actinomycetes* increase the remarkable catabolic versatility, shown within the *phylum*, where some species of *Nocardia* and *Gordonia* are well known for degrading high–molecular-weight polymers and long-chain or aromatic hydrocarbons [76,79]. For example, in *Gordonia rubripertincta* CWB2, the PAL ortholog gru-GCWB2_23930 is reported to support a ‘hybrid’ styrene-degradation pathway that feeds into the PAA route [80]. Other *Actinobacteria*, for which experimental support for a functional PAA pathway exists, include *R. jostii* RHA1 [15] and *Corynebacterium glutamicum* AS 1.542, which carries a complete PAA cluster regulated by PaaR [75]. Additional *Corynebacterium* species likewise degrade a wide range of hydrocarbons [81]. In *Streptomyces*, CoA-thioester activation of aromatic substrates [82] and the identification of a PaaE-type oxidoreductase in *Streptomyces peucetius* [83] further support the presence of functional, though often remodelled, PAA pathways across *Actinomycetes* in this clade.

## Clade 4

### Stability of the PAA pathway in *Gammaproteobacteria* and remodelling across bacterial and archaeal lineages

This clade spanned into three major subclades (Fig. 5). Two of them (G3 and G4) comprised PALs predicted in *Gammaproteobacteria*, which show the most homogeneous FP pathway distribution, whereas a third subclade clusters PALs located outside of *Proteobacteria* (now *Pseudomonadota*).

Subclade G3 includes marine and environmental genera (*Amphritea*, *Halomonas*, *Marinobacter*, *Marinomonas*, *Psychromonas*, *Shewanella* and *Pseudomonas*). Most strains retain a stable FP configuration regulated by PaaX, although some lineages exhibit UP–PaaX arrangements or loss of PaaY. Plasmid-borne PALs occur only in isolated cases. Overall, G3 represents a functionally conserved group with limited pathway remodelling.

Subclade G4 comprises *Acinetobacter* and *Zobellella* (G4a) together with multiple *Enterobacterales* lineages (G4b–e) (Fig. 5). In G4a, PALs predominantly occur in FP configurations with PaaR–X regulation and the retention of PaaY, although some *Acinetobacter* strains exhibit FP–X–Y or UP–X–Y configuration. It is reported that the PAA pathway in *Acinetobacter baumannii* is induced by antibiotics, oxidative stress and diverse environmental cues, including pleural fluid, antibiotics such as imipenem and cephalosporins and exposure to blue light, and has been linked to biofilm formation and virulence [84,85]. Similar roles have been reported in *B. cenocepacia*, where the inactivation of *paa* genes reduces this pathogen’s virulence in *Caenorhabditis elegans* [7].

The identification of a complete *paa* cluster in *Zobellella denitrificans* F13-1 is notable, as this genus is primarily known for denitrification in saline, organic-rich environments [86]. Its PAA pathway may enable the processing or detoxification of aromatic compounds in such habitats, a function not previously reported for this genus [87].

*Enterobacterales* largely retain FP architectures but display extensive regulatory diversification, including PaaX, PaaR–X and TF-absent states, with recurrent loss of PaaY in several genera. Although some PALs are plasmid-encoded, most remain chromosomal, reflecting increased pathway fragmentation and regulatory reorganization. In clinically relevant genera such as *Klebsiella* and *Raoultella*, conservation of the *paa* cluster, typically regulated by PaaX, supports roles in survival under aromatic-rich and stress-prone environments, including clinical settings [30,88,90].

Unusual configurations lacking local regulators in genera, such as *Cedecea*, suggest evolutionary histories shaped by horizontal gene transfer and recurrent regulatory loss. Given the association of the PAA pathway with virulence and biofilm formation across multiple pathogens, these patterns support a broader role for the pathway as a metabolic–regulatory hub that integrates aromatic metabolism, stress responses and niche adaptation [7].

### Beyond *Gammaproteobacteria*

Outside *Enterobacterales*, PALs in *Alphaproteobacteria*, including *Komagataeibacter* and two *Rhodopseudomonas* paralogs (subclade AlpC), display reduced or UP configurations with variable regulatory content. In parallel, diverse bacterial and archaeal lineages exhibit repeated remodelling of partial epoxidation modules and transcriptional regulators, as observed in *Bacilli*, *Clostridia* and *Deinococci*, as well as through the acquisition of FP configurations in *Flavobacteriia* and in two *Hymenobacter* strains (*Cytophagia*). Together, these patterns underscore the pronounced evolutionary plasticity of the PAA pathway across domains.

### Co-evolution of genomic markers

The 1,2-phenylacetyl-CoA epoxidase multicomponent enzyme subunits PaaA and PaaC have been proposed in previous studies as robust genomic markers of the PAA pathway, owing to their broad conservation across taxonomic groups [8]. Consistent with this view, our analysis shows that these subunits are retained in all 825 proteomes that encode a PAL enzyme. This widespread conservation prompted us to ask whether PaaA and PaaC have followed shared evolutionary trajectories, as expected for functionally coupled components of the same metabolic module.

To address this question, we performed a cophylogenetic analysis of PaaA and PaaC from 219 representative taxa using a PACo. This framework provides quantitative measures of phylogenetic congruence between the two primary sequence phylogenies and identifies individual associations that either support or deviate from a shared evolutionary history represented as residual values (Table S9).

**Fig. 6.**
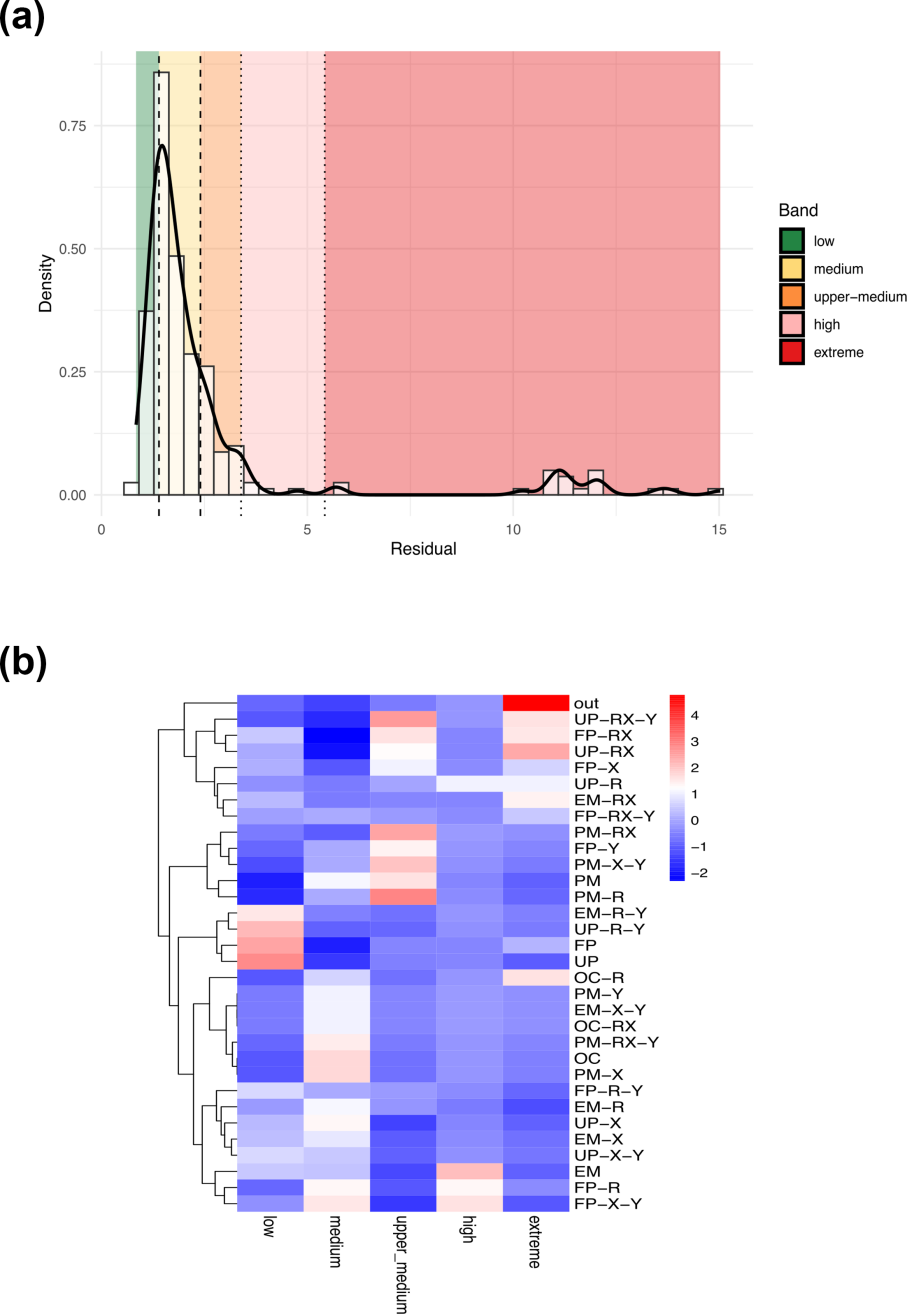
Co-evolutionary signal between PaaA and PaaC across pathway configurations. (**a**) Distribution of PACo residuals from the cophylogenetic analysis of PaaA and PaaC. The histogram and density curve show a strongly right-skewed distribution, with most associations exhibiting low residual values consistent with codivergence. Vertical dashed and dotted lines indicate quantile-based thresholds defining residual bands (low to extreme). (**b**) Heatmap of standardized *χ*² residuals showing the association between pathway configurations and PACo residual classes. Red and blue cells indicate over- and under-representation, respectively, relative to expectations under independence.

The histogram of PACo residuals shown in Fig. 6a exhibits a pronounced right-skewed distribution, with most associations clustered at low residual values. Low residuals correspond to well-fitting links, consistent with local codivergence between PaaA and PaaC, whereas higher residuals indicate phylogenetic incongruence. Such incongruence may arise from processes including host switching, paralogy or recombination, rate heterogeneity or, in some cases, labelling or mapping errors.

Applying quantile-based thresholds allowed the residuals to be grouped into biologically interpretable categories (Table S9). Residuals between the first and third quartiles (Q1=1.40 to Q3=2.40) represent moderate departures from perfect congruence, whereas values above the 90th percentile (p90=3.39) reflect increasingly divergent evolutionary trajectories. A small but clearly defined subset of associations exceeds the extreme threshold (Q3+3×IQR≈5.42), forming a distinct tail of outliers. These cases likely correspond to decoupled evolutionary histories, potentially driven by horizontal gene transfer, gene replacement or lineage-specific remodelling of the pathway.

Overall, this distribution indicates that heterogeneity in PACo residuals is largely driven by a limited number of extreme associations, while the vast majority of PaaA–PaaC pairs retain a strong co-evolutionary signal. This pattern supports the use of quantile-based bands to distinguish stable, co-evolving pathway architectures from a smaller set of highly divergent evolutionary events. Importantly, these patterns are unlikely to reflect biases in dataset composition, as the analysis was based on a curated subset of representative genomes that preserves phylogenetic, functional and environmental diversity, including all major pathway configurations (FP, UP, EM, PM and OC).

Consistent with these results, the tanglegram tree shows a high degree of topological congruence between the PaaA and PaaC phylogenies, with most links running approximately parallel to one another and remaining within corresponding clades (Fig. S8). This pattern indicates broadly shared evolutionary trajectories for the two subunits and is consistent with their long-term functional coupling and co-inheritance as part of a conserved metabolic module. Congruent regions are particularly evident within major bacterial lineages, including *Alphaproteobacteria* (AlpA, AlpC, AlpD and AlpE), *Gammaproteobacteria*, *Betaproteobacteria*, *Actinomycota* and *Bacillota* (previously known as *Firmicutes*), where clade-specific groupings are preserved in both trees. In contrast, several PaaA–PaaC pairs within *Alphaproteobacteria*, particularly in the AlpF-a and AlpF-b groups, display medium to high residuals, including some extreme values, suggesting weaker or disrupted co-evolutionary histories in these lineages.

To test whether co-evolutionary patterns are associated with pathway organization, we evaluated the relationship between PACo residual classes and pathway configuration using Cramér’s V (Fig. S9). This analysis revealed a strong association between configuration and residual category (bias-corrected Cramér’s V=0.41). Although the *χ*² test provided only marginal support (*P*=0.059), a permutation-based test of independence confirmed a statistically significant association (*P*=0.036), indicating that residual categories are non-randomly structured by pathway configuration (Fig. 6b).

## Concluding remarks

By integrating conserved motif signatures with protein architecture recognition, genomic context analysis, structural modelling, phylogenetics and cophylogenetic inference, this study provides a comprehensive evolutionary framework for PALs and the PAA pathway. We showed that PALs form a structurally and mechanistically conserved enzyme family, whose catalytic core has been maintained across broad taxonomic distances despite extensive sequence divergence. Robust conservation of key motifs and the adenylate-forming fold supports the reliability of our PAL predictions and confirms strong evolutionary constraints on phenylacetate activation.

Our analyses reveal that the PAA pathway is highly dynamic, shaped by recurrent gene gain, loss and duplication, as well as regulatory remodelling, across bacteria and archaea. While *Gammaproteobacteria* likely represent the ancestral origin of the pathway, *Betaproteobacteria* emerge as the principal hotspot of diversification, exhibiting extensive paralogous redundancy, replicon shuffling and pathway fragmentation. In contrast, *Alphaproteobacteria*, *Actinomycetes* and *Firmicutes* display lineage-specific patterns ranging from relatively conserved full pathways to highly reduced or modular configurations, reflecting distinct ecological strategies and genome architectures.

Paralogous redundancy of PAL and other aryl-CoA ligases appears to be a key adaptive feature, enhancing metabolic flexibility, detoxification capacity and fitness under fluctuating environmental conditions. This redundancy is frequently associated with differential replicon localization and regulatory control, supporting a model in which megaplasmids and secondary chromosomes act as reservoirs for functional backup and innovation.

Finally, cophylogenetic analyses demonstrate that the epoxidase subunits PaaA and PaaC have largely co-evolved as a tightly coupled module, validating their use as robust genomic markers of the PAA pathway. Deviations from this co-evolutionary signal are limited and correlate with specific pathway configurations, highlighting discrete evolutionary events such as horizontal transfer or pathway remodelling.

Collectively, our findings establish the PAA pathway not merely as a catabolic route for aromatic hydrocarbons, but as an evolutionarily flexible metabolic–regulatory hub that integrates environmental sensing, stress responses and niche adaptation, with implications for hydrocarbon degradation, microbial competition and pathogenicity.

Finally, beyond these insights, the integrative framework developed here (combining protein architecture recognition, motif conservation and genomic context analysis) provides a robust strategy for detecting PAA pathway components in complex genomic datasets. In particular, the identification of conserved PAL-associated motifs offers a sensitive and scalable approach for exploring PAA metabolic potential in metagenomes and MAGs, where gene fragmentation and incomplete assemblies often hinder functional annotation. This framework, therefore, opens new avenues for tracking aromatic hydrocarbon degradation capacity across diverse, uncultured microbial communities.

## Supplementary material

10.1099/mgen.0.001719 Uncited Supplementary Material 1.

10.1099/mgen.0.001719 Uncited Supplementary Material 2.

## Abbreviations

AHs: aromatic hydrocarbons
ANL: adenylate-forming enzyme
BCL: benzoate-CoA ligase
EM: complete epoxidase multicomponent
FP: full pathway
GCO: genomic context organization
iTOL: Interactive Tree of Life
KEGG: Kyoto Encyclopedia of Genes and Genomes
LW: lower pathway
MAGs: metagenome-assembled genomes
PAA: phenylacetic acid
PACo: Procrustean Approach to Cophylogeny
PACoA: phenylacetyl-CoA
PAL: phenylacetate-CoA ligase
RMSD: root mean square deviation
TFs: transcriptional regulators
UP: upper pathway
