# Supplementary material for: Conservation, origin, and remodeling of the phenyl acetate degradation pathway
Source: Microb Genom. 2026 May 19;12(5):001719. doi: 10.1099/mgen.0.001719 (PMC13186573; doi:10.1099/mgen.0.001719)
Supplement: Uncited Supplementary Material 1. [file mgen-12-01719-s001.pdf]

Supplementary Table Legends

Table S1. Experimentally validated PAA pathway-updated genomes. This table presents an updated list of genomes for which the phenylacetic acid (PAA) degradation pathway has been previously reported and experimentally validated, incorporating revised genome annotations and current pathway reconstructions.

Table S2. PAA pathway reannotation. The table lists PAA pathway–related sequences recovered by BlastX searches that were previously annotated as pseudogenes or identified through manual inspection of the corresponding genomic contexts.

Table S3. Non-redundant PAL sequences. CD-HIT clustering and the representative sequences selected for motif analysis using MEME, as described in the Methods section.

Table S4. Mast results. MEME motifs identified by MAST in AMP-binding and AMP-binding_C2 PAL sequences, preserving the motif arrangement proposed for the identification of PAL proteins.

Table S5. PAL Paralogs. The distribution of PAL paralogous copies and the GCO identity is shown. Taxonomic assignment and replicon location are also shown.

Table S6. Structural comparison of predicted PAL models against the reference structure. Local similarity was measured as RMSD of Cα atoms after optimal superposition and the number of aligned residues. Global similarity was assessed using TM-score and associated RMSD values for each predicted model.

Table S7. Distribution, prediction, and curation of PAA pathway enzymes across genomes. The table summarizes the curated annotation of PAA pathway

enzymes identified within their GCO. For each genome, the presence and organization of pathway enzymes are explicitly reported, together with taxonomic assignment, replicon location, and clade classification. In addition, for each phenylacetate-CoA ligase (PAL) gene, the MAST e-value associated with its motif composition is provided.

Table S8. Features of PAL sequences used in the pruned phylogenetic tree. This table lists the PAL sequences selected to construct the pruned phylogenetic tree. For each sequence, taxonomic assignment, replicon location, pathway configuration, clade classification, and genus membership are provided.

Table S9. Residual values. Residual values obtained for each PaaA-PaaC pair are listed in the tables. Residuals are organized by codivergence value. Additionally, the isolation site and, when available, a brief description of the physiological characteristics.

Supplementary figure legends

Figure S1. Multiple sequence alignment of PAL candidates showing conservation of the PAL-2 motif. The glycine-rich "GLG" sequence, shared with benzoate-CoA ligases (BCLs), is conserved across PALs. Notably, the alanine residue located two positions upstream of this motif in BCLs (Ala227 in *Rhodopseudomonas palustris*) is consistently replaced by a conserved threonine in PAL sequences analyzed in this study.

Figure S2. Genomic organization of PAA pathway components. Core epoxidation genes (*paaA–paaC*) and the regulator *paaX* are largely restricted to the PAL genomic context, whereas other PAA-associated enzymes are widely distributed across genomes, reflecting differences in domain specificity.

Figure S3. PAL structural superposition of predicted models with the crystallographic reference. The figure highlights the conservation of the catalytic core and the variability of the peripheral and accessory domains.

Figure S4. Structural prediction and comparison of phenylacetate-CoA ligase proteins. The figure shows the superposition of 13 selected PAL ortholog and paralog structural models onto the crystallographic reference structure from *Burkholderia cenocepacia* J2315 (PDB ID: 2Y4N, chain B), shown in pink. Structural similarity was assessed using optimal Cα-atom superposition, enabling direct comparison of conserved core regions across models. The overlay highlights a high degree of structural conservation among PAL variants despite sequence divergence, particularly within the catalytic core and substrate-binding regions, supporting their functional equivalence.

Figure S5. Distribution of PAL across microbial phyla. The bar plot shows the taxonomic distribution of PAL among bacterial and archaeal phyla.

Figure S6. Phylogenetic organization of PAL and PAA pathway configurations. The tree depicts the evolutionary relationships among 853 PAL enzymes, with associated pathway configurations indicated by colored symbols on the right. Replicon localization of each pathway is also shown. Bootstrap support values are indicated at the corresponding nodes.

Figure S7. Phylogenetic organization of PAL-genus representatives and PAA pathway configurations. The tree depicts the evolutionary relationships among 186 PAL-genus enzymes, with associated pathway configurations indicated by colored symbols on the right. Replicon localization of each pathway is also shown. Bootstrap support values are indicated at the corresponding nodes.

Figure S8. Cophylogenetic relationships between PaaA and PaaC and their association with pathway configuration and PACo residual classes. Tanglegram showing the phylogenetic trees of the PaaA and PaaC subunits of the multicomponent phenylacetyl-CoA oxygenase, connected by links representing inferred associations between paired sequences. Taxa are colored according to their major phylogenetic classes. Adjacent annotation tracks indicate pathway configuration, PAA-based configuration classes, and PACo residual categories (low, medium, upper-medium, high, extreme). Red links highlight associations that disproportionately contribute to high and extreme PACo residuals, indicating divergent or decoupled evolutionary histories, whereas black links correspond to low-, medium-, and upper-medium residual associations, consistent with parallel evolution.

Figure S9. Association between pathway configuration and PACo residual class. Heatmap showing the strength of association between pathway configurations and PACo residual classes, quantified using bias-corrected Cramér's V. Rows correspond to pathway configurations and columns to residual categories (low to extreme). Color intensity reflects increasing association strength, with higher values indicating stronger non-random associations between specific configurations and residual classes.

Figure S1

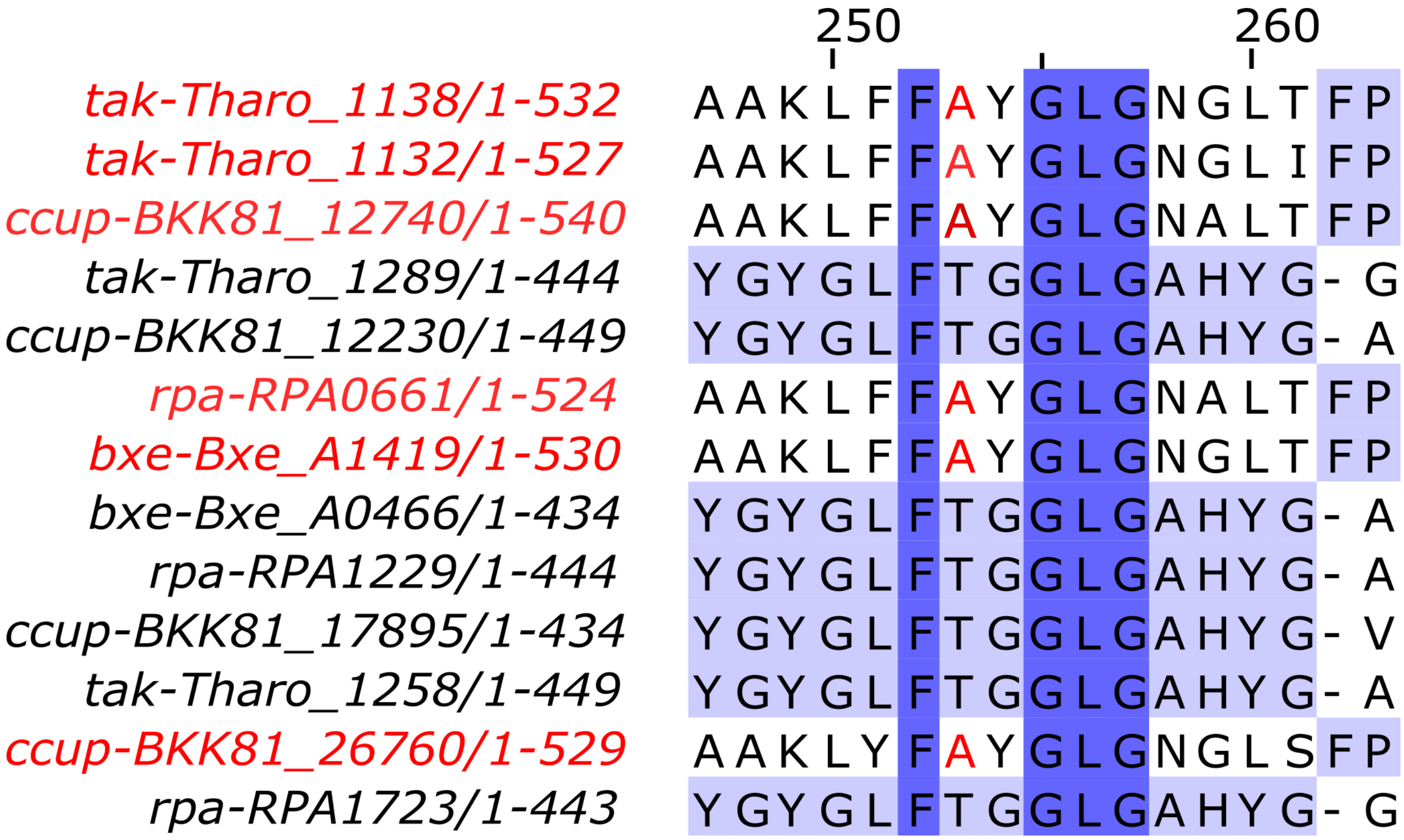

250
260
tak-Tharo_1138/1-532 AAKLFFAYGLGNGLTFP
tak-Tharo_1132/1-527 AAKLFFAYGLGNGLIFP
ccup-BKK81_12740/1-540 AAKLFFAYGLGNALTFP
tak-Tharo_1289/1-444 YGYGLFTGGLGAHYG-G
ccup-BKK81_12230/1-449 YGYGLFTGGLGAHYG-A
rpa-RPA0661/1-524 AAKLFFAYGLGNALTFP
bxe-Bxe_A1419/1-530 AAKLFFAYGLGNGLTFP
bxe-Bxe_A0466/1-434 YGYGLFTGGLGAHYG-A
rpa-RPA1229/1-444 YGYGLFTGGLGAHYG-A
ccup-BKK81_17895/1-434 YGYGLFTGGLGAHYG-V
tak-Tharo_1258/1-449 YGYGLFTGGLGAHYG-A
ccup-BKK81_26760/1-529 AAKLYFAYGLGNGLSFP
rpa-RPA1723/1-443 YGYGLFTGGLGAHYG-G

Figure S2

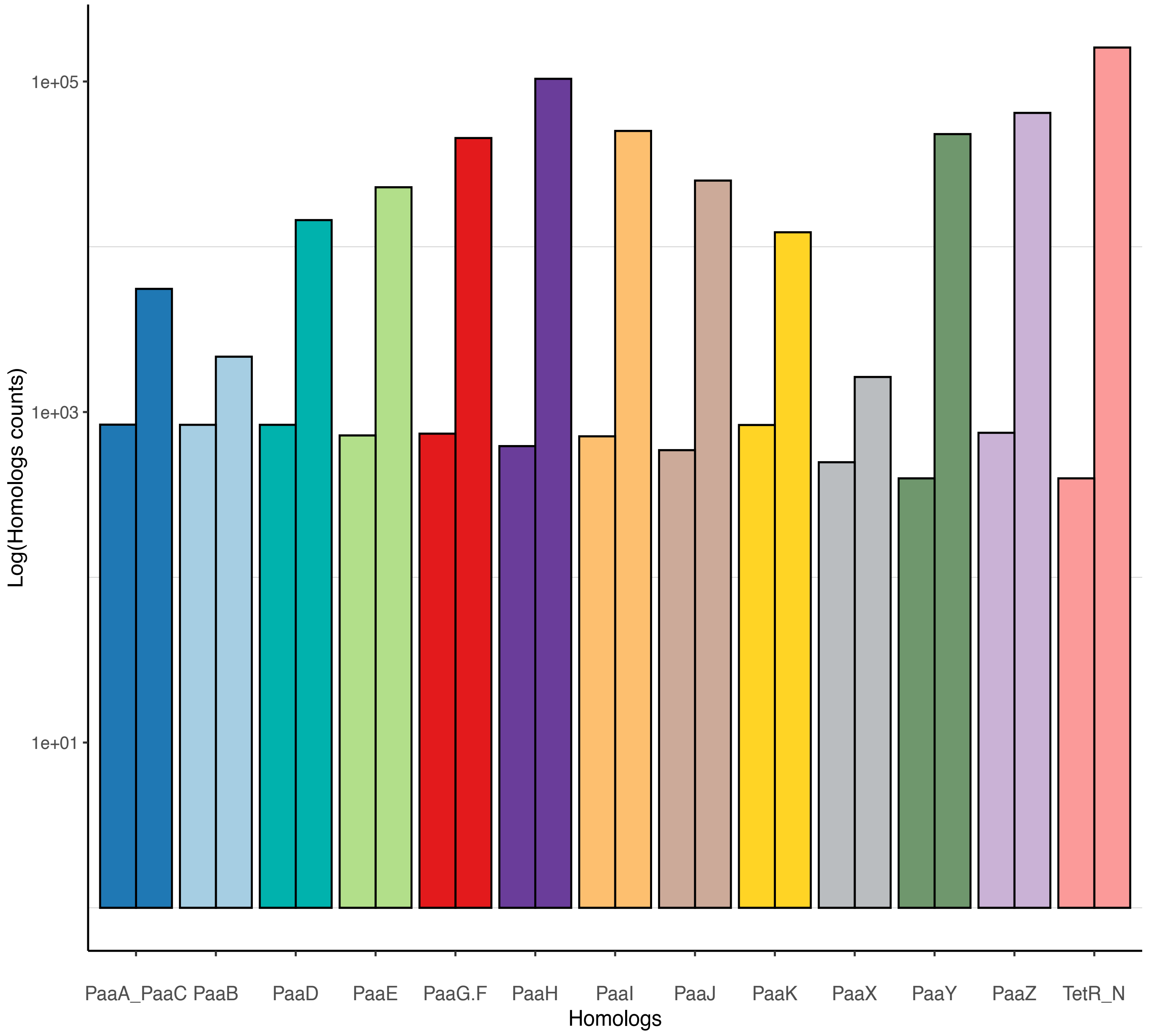
1e+05
1e+03
1e+01
Log(Homologs counts)
PaaA_PaaC
PaaB
PaaD
PaaE
PaaG.F
PaaH
PaaI
PaaJ
PaaK
PaaX
PaaY
PaaZ
TetR_N
Homologs

Figure S3

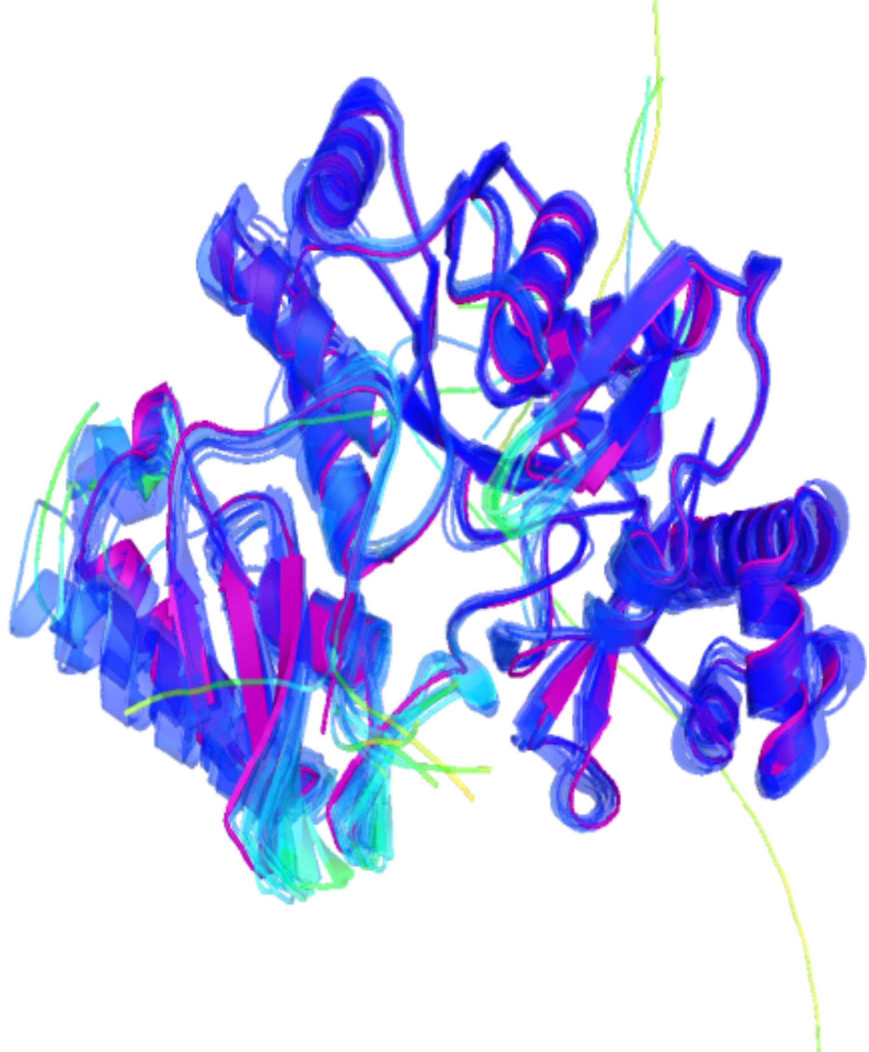

Figure S4

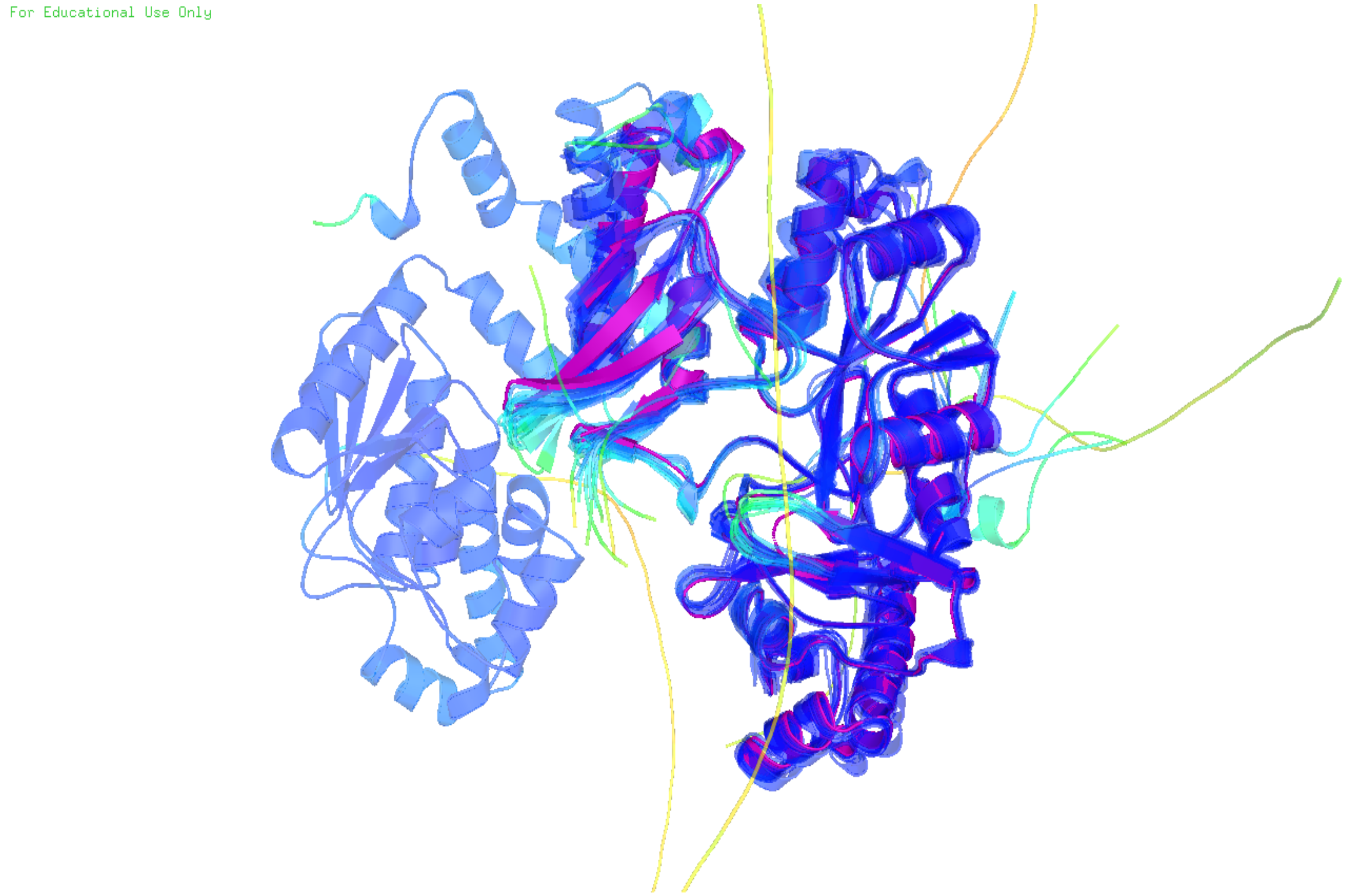

Figure S5

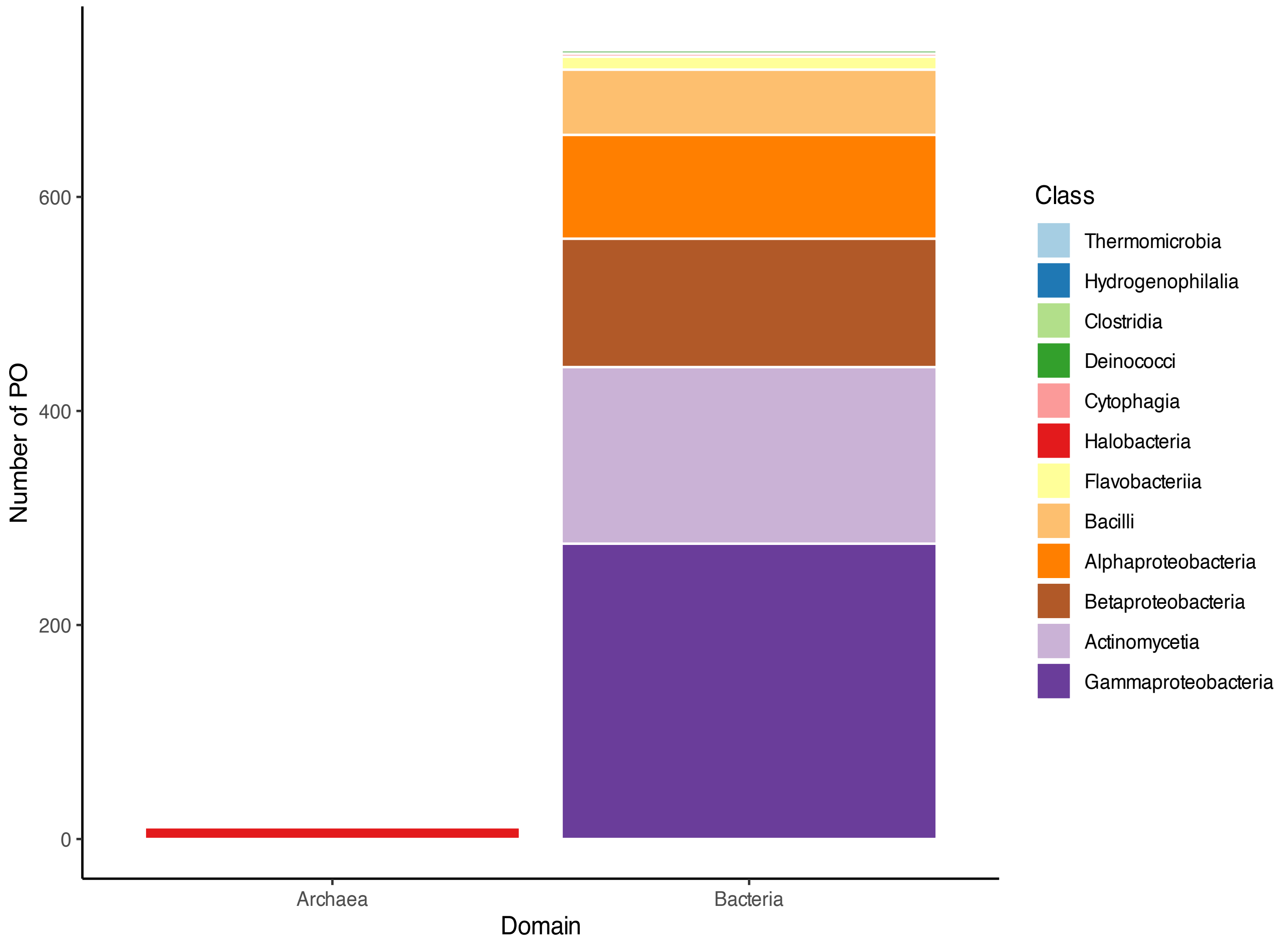

Number of PO
600
400
200
0
Archaea
Bacteria
Domain
Class
Thermomicrobia
Hydrogenophilalia
Clostridia
Deinococci
Cytophagia
Halobacteria
Flavobacteriia
Bacilli
Alphaproteobacteria
Betaproteobacteria
Actinomycetia
Gammaproteobacteria

Figure S6

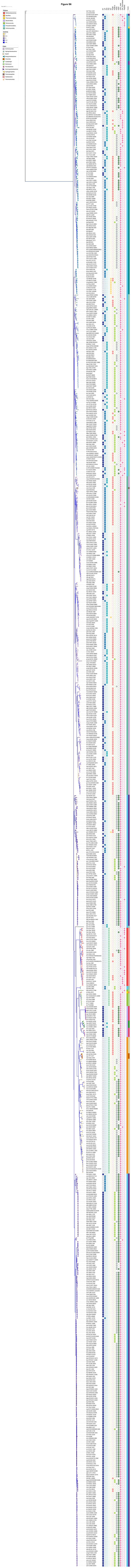

Figure S7

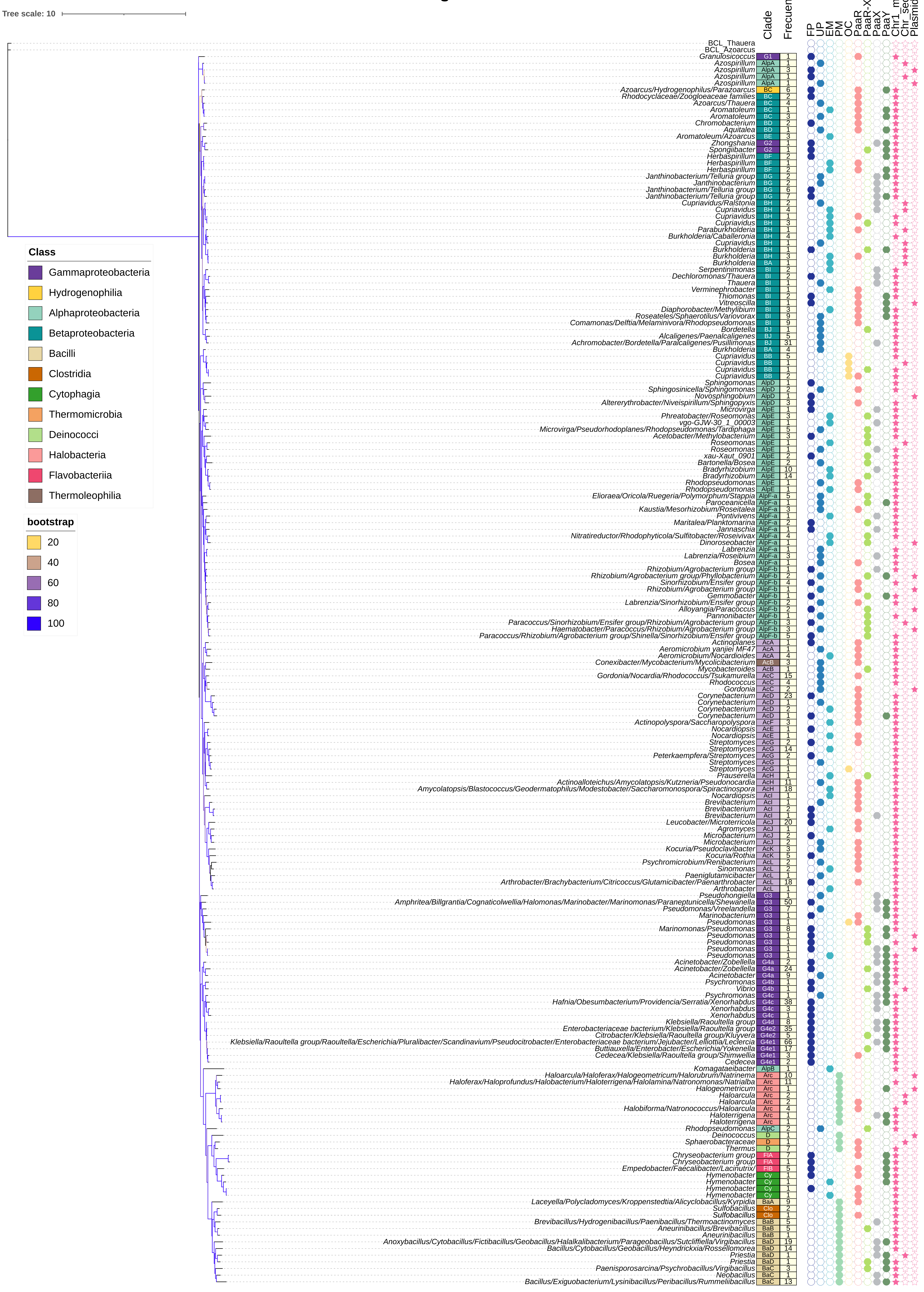

Tree scale: 10
Clade
Frecuency
FP
UP
EM
PM
OC
PaaR
PaaR-X
PaaX
PaaY
Chr1_main
Chr_secondaries
Plasmid
BCL_Thauera
BCL_Azoarcus
Class
Gammaproteobacteria
Hydrogenophilia
Alphaproteobacteria
Betaproteobacteria
Bacilli
Clostridia
Cytophagia
Thermomicrobia
Deinococci
Halobacteria
Flavobacteriia
Thermoleophilia
bootstrap
20
40
60
80
100

Figure S8

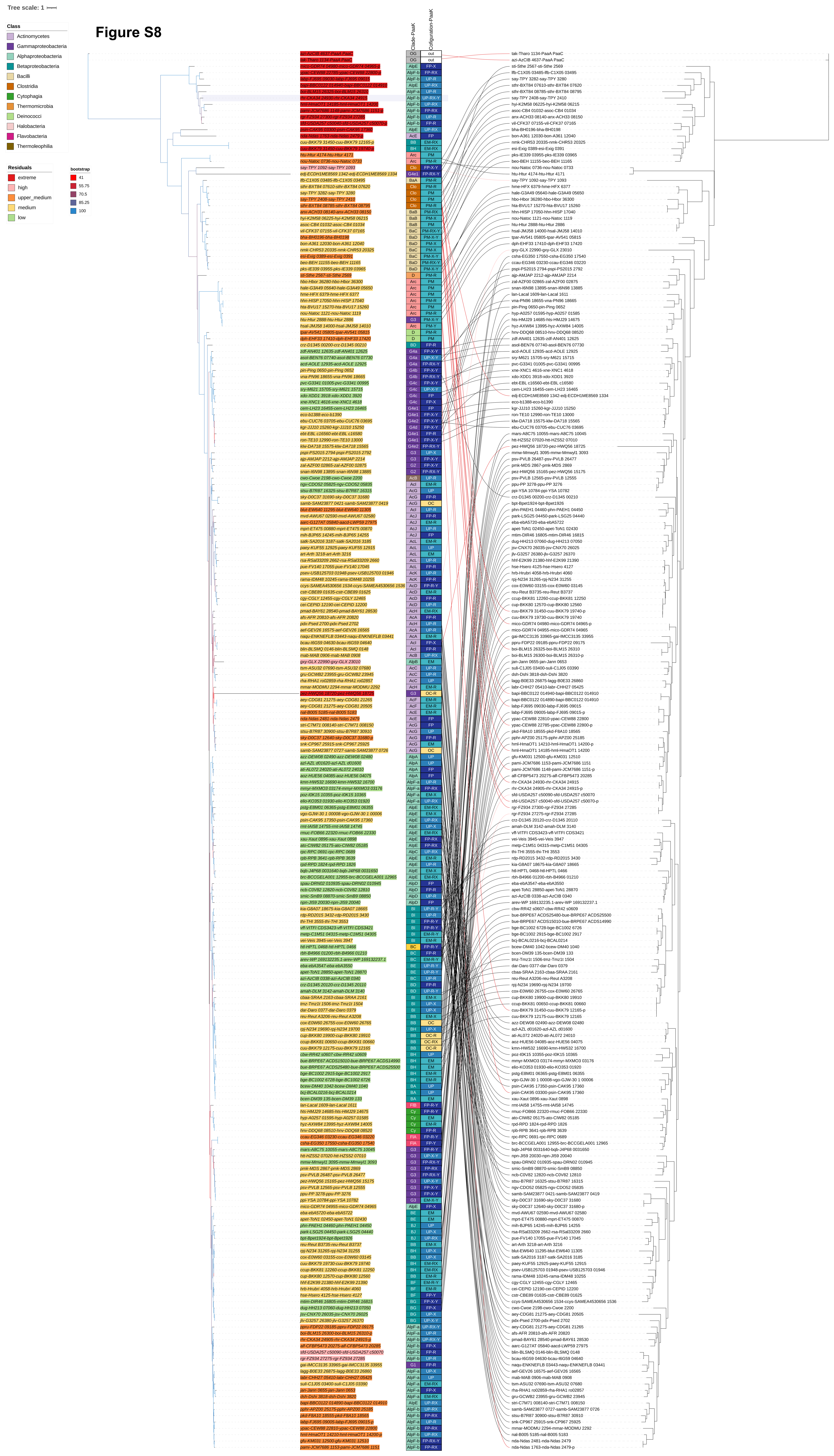

Tree scale: 1
Class
Actinomycetes
Gammaproteobacteria
Alphaproteobacteria
Betaproteobacteria
Bacilli
Clostridia
Cytophagia
Thermomicrobia
Deinococci
Halobacteria
Flavobacteria
Thermoleophilia
Residuals
extreme
high
upper_medium
medium
low
bootstrap
41
55.75
70.5
85.25
100
Clade-PaaK
Configuration-PaaK

Figure S9

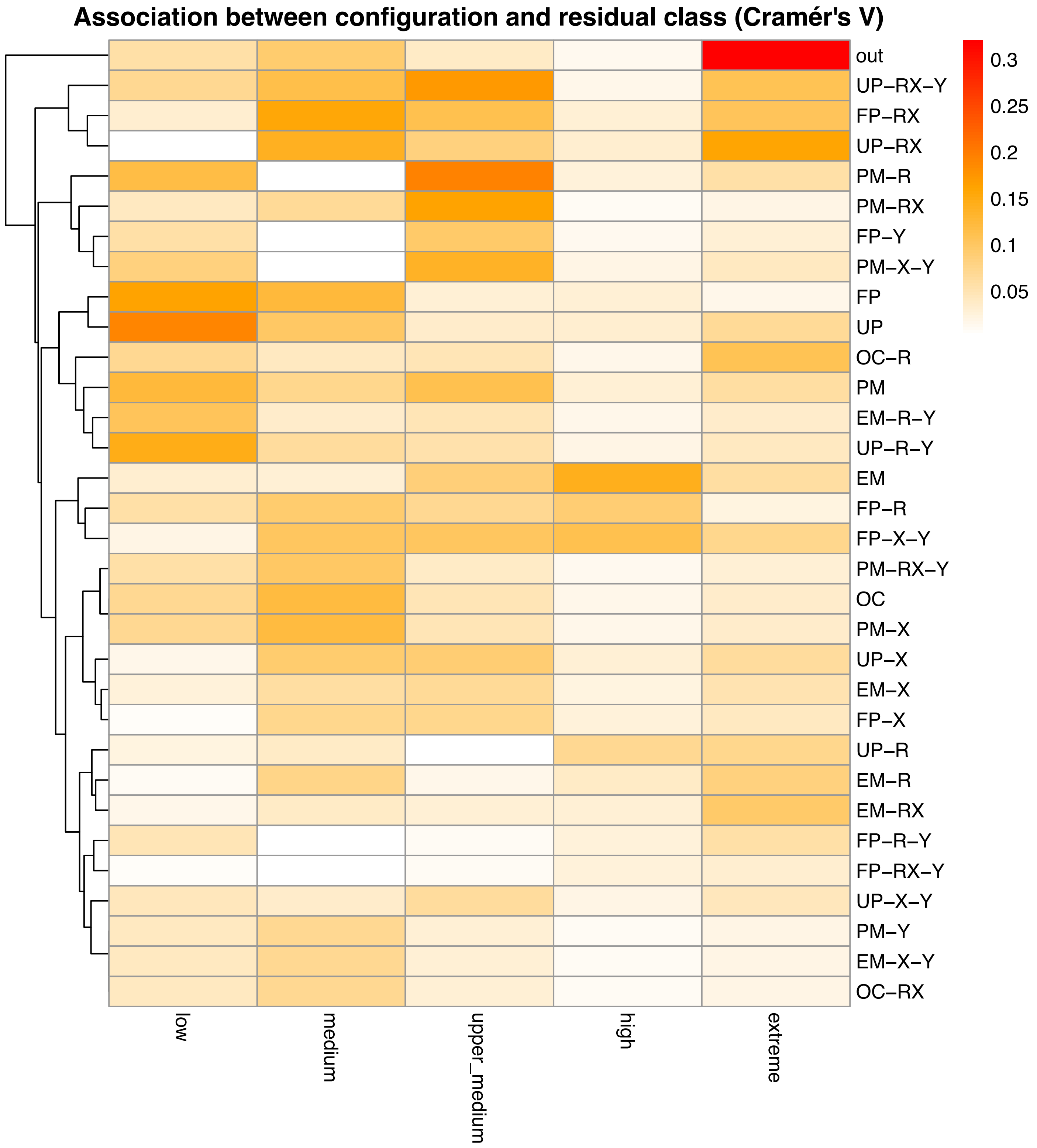

Association between configuration and residual class (Cramér's V)
out
UP−RX−Y
FP−RX
UP−RX
PM−R
PM−RX
FP−Y
PM−X−Y
FP
UP
OC−R
PM
EM−R−Y
UP−R−Y
EM
FP−R
FP−X−Y
PM−RX−Y
OC
PM−X
UP−X
EM−X
FP−X
UP−R
EM−R
EM−RX
FP−R−Y
FP−RX−Y
UP−X−Y
PM−Y
EM−X−Y
OC−RX
low
medium
upper_medium
high
extreme
0.3
0.25
0.2
0.15
0.1
0.05
